# The Acute and Chronic Cognitive and Cerebral Blood-Flow Effects of Nepalese Pepper (*Zanthoxylum armatum* DC.) Extract—A Randomized, Double-Blind, Placebo-Controlled Study in Healthy Humans

**DOI:** 10.3390/nu11123022

**Published:** 2019-12-10

**Authors:** David Kennedy, Emma Wightman, Julie Khan, Torsten Grothe, Philippa Jackson

**Affiliations:** 1 Brain, Performance and Nutrition Research Centre, Northumbria University, Newcastle-upon-Tyne NE1 8ST, UK; emma.l.wightman@northumbria.ac.uk (E.W.); julie.khan@northumbria.ac.uk (J.K.); philippa.jackson@northumbria.ac.uk (P.J.); 2 Mibelle Group Biochemistry, Bolimattstrasse 1, CH-5033 Buchs, Switzerland; torsten.grothe@mibellegroup.com

**Keywords:** Nepalese pepper, cognition, cerebral blood flow, hydroxy-α-sanshool

## Abstract

*Background*: *Zanthoxylum armatum* DC. (ZA) is a traditional Asian culinary spice and medicinal compound, which is rich in monoterpenes and hydroxy α-sanshool. Mechanistic interactions with the monoamine, cholinergic and cannabinoid neurotransmission systems, as well as transient receptor potential (TRP) and potassium ion channels, may predispose ZA to modulate human brain function. *Objectives*: To investigate the effects of a single dose and 56-days supplementation with a lipid extract of ZA on cognitive function, mood and cerebral blood-flow (CBF) parameters in the pre-frontal cortex during cognitive task performance. *Design*: Double-blind, randomized, parallel groups study with N = 82 healthy males and females between the ages of 30 and 55 years. Assessments were undertaken pre-dose and at 1, 3 and 5 h post-dose on the first (Day 1) and last (Day 56) days of supplementation. *Results*: A single dose of ZA (Day 1) resulted in acute improvements on a ‘Speed of Attention’ factor and the Rapid Visual Information Processing (RVIP) task, in comparison to placebo. However, following ZA participants were less accurate on the name-to-face recall task. After 56 days of ZA consumption (Day 56), speed was enhanced on a global ‘Speed of Performance’ measure, comprising data from all of the timed tasks in the computerized battery. Participants also completed more correct Serial 3s Subtractions at the 3 h assessment and were less mentally fatigued throughout the day than participants consuming placebo. These effects were complemented on both Day 1 and Day 56 by modulation of CBF parameters, as assessed by Near Infrared Spectroscopy (NIRS). The primary finding here was a reduced hemodynamic response during the RVIP task. *Conclusion*: ZA improves aspects of cognitive performance, in particular the speed of performing tasks, in healthy humans and results in concomitant reductions in hemodynamic responses in the frontal cortex during task performance. The findings suggest an increase in neural efficiency following ZA.

## 1. Introduction

The plant species *Zanthoxylum armatum* DC. (ZA; also known as Nepalese or Timur pepper) is from the *Rutaceae* or citrus, family and its bark, seeds and fruits have a long history as sources of medicinal treatments. This is particularly prevalent in Chinese, Nepalese and Ayurvedic medical systems with indications including as a treatment for depression, gastro-intestinal and digestive disorders and as a topical treatments for toothache and skin irritation/wounds [1,2,3]. It is also one of several species of the *Zanthoxylum* genus generically known as Szechuan pepper; a traditional Chinese and Nepalese culinary spice comprising the fruit pericarp which is typically added to wok and dumpling dishes to provide a characteristic grapefruit-like taste and flavor and a tingling numbness in the mouth.

**Figure 1:**
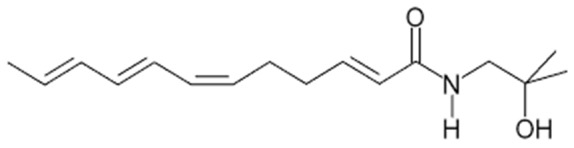
Chemical structure of hydroxy α-sanshool.

Whilst the various tissues from ZA have complex phytochemistry, the tingling mouth sensation, and any gastro-intestinal or dental applications, are related to the presence of a number of alkamides and unsaturated fatty acid amides; most notably hydroxy α-sanshool (see Figure 1) and related structures [4]. The alkamide group of compounds, which are also found in 300+ species from the *Zanthoxylum*, *Acmella (Spilanthes)*, *Anacyclus*, *Echinacea* and *Heliopsis* genera, typically also have insecticidal, larvicidal and antimicrobial properties and act as plant growth factors [4]. In mammals, they have anti-inflammatory, immunomodulatory and analgesic properties and may underlie the putative medicinal effects of plants such as Echinacea and ‘maca’ (*Lepidium meyeni*) [1,4]. Evidence suggests that alkamides can readily cross the skin, oral and gut mucosa and that they can traverse the blood brain barrier [5]. In rodent models, the alkamide riparin II has been shown to reduce anxiety [6] and attenuate depression via monoaminergic mechanisms [7].

In a medicinal context, ZA is most likely to be encountered as a component of the traditional Japanese herbal medicine Daikenchuto, which comprises ginger rhizome, ginseng root, rice gluten and *Zanthoxylum* fruit. Daikenchuto is generally indicated for gastrointestinal disorders [8], with some evidence of efficacy following abdominal surgery [9,10]. In a study assessing the pharmacokinetics of Daikenchuto, it was demonstrated that hydroxy α-sanshool achieved by far the highest plasma levels of the six compounds measured. Its bioavailability peaked after 30 min with a mean half-life of approximately 2 h [11]. A single study [12] demonstrated that Daikenchuto, administered for 3 days, improved mouse learning and memory and attenuated the cognitive decrements associated with the muscarinic receptor antagonist scopolamine. The learning and memory effects were also evident following both the ZA and hydroxy α-sanshool components by themselves, confirming that hydroxy α-sanshool was the active component in terms of learning and memory benefits.

In mechanistic terms, the gastrointestinal effects of Daikenchuto have been attributed to increased acetylcholine release [13]. In line with this, an attenuation of the learning and memory decrements associated with scopolamine in rodents suggests cholinergic activity as a potential mechanism of any brain function effects of Daikenchuto and its active ingredients [12]. However, it is also notable that alkamides such as γ-sanshool have been shown to interact with cannabinoid CB_1_ and CB_2_ receptors; due to the structural similarity between the sanshool group and endocannabinoids [14]. In general, the oral sensations associated with a variety of spices and food additives are attributable to interactions with differing transient receptor potential (TRP) channels. These plasma membrane ion channels modulate the entry of positively charged ions into numerous cell types and mediate the perception of sensations such as pressure, temperature and pain [15]. The tingling numbness associated with hydroxy α-sanshool is specifically due to interactions with TRPV1 (also known as the vanilloid receptor) and TRPA1 receptors in sensory neurons, which result in an influx of Ca^2+^ [16]. Hydroxy α-sanshool also inhibits members of the two-pore domain potassium ion channel family (designated as the KCNK family by the Human Genome Organization) that are sensitive to mechanical stimuli, pH, signaling lipids and other G-protein receptor ligands including neurotransmitters and anesthetics. In this regard hydroxy α-sanshool specifically inhibits family members TASK-1 (TWIK-related acid-sensitive potassium-1), TASK-3 and TRESK [4]. Interestingly, evidence shows that both the TRP and KCNK ion channels that are modulated by hydroxy α-sanshool are widely distributed in cognition relevant brain regions [17,18].

Alongside significant levels of monoterpenes, which have been shown to exert independent beneficial effects on cognitive function [19,20], it is possible that an extract rich in hydroxy α-sanshool will have multifarious effects on human brain function. The current double-blind, randomized, parallel groups study therefore investigated the effects of a single 80 mg dose and 56-days supplementation with a lipid extract of ZA on cognitive function, mood and cerebral blood-flow parameters in the frontal cortex.

## 2. Methods

### 2.1. Design

The study adopted a double-blind, placebo-controlled, parallel-groups design, in which the acute and chronic effects of *Zanthoxylum armatum* DC. (Nepalese pepper) extract (ZA) and placebo were assessed pre-dose and at 1, 3 and 5 h post-dose on the first day (i.e., acute effects) and following 56 days (+/−3 days) consumption of the intervention. An optional, concomitant investigation of the cerebral blood-flow effects of the extract was carried out in ~50% of the total sample.

### 2.2. Participants

The final sample comprised a total of 82 participants, aged 30 to 55 years, who self-reported themselves to be in good health. Participants were excluded from the study if they: Had any pre-existing medical condition/illness or were currently taking prescription medications which might have an impact on their ability to take part in the study; were taking any dietary supplements including omega fatty acids/fish oils; had high blood pressure (systolic over 159 mm Hg or diastolic over 99 mm Hg); had a Body Mass Index (BMI) outside of the range 18–35 kg/m^2^; were pregnant, seeking to become pregnant or lactating; had learning difficulties or dyslexia; had an uncorrected visual impairment; were smokers or regular consumers of nicotine containing products; had a history of alcohol or drug abuse; consumed caffeine in excess of 500 mg per day; had any food intolerances/sensitivities; were unable to complete all of the study assessments or were non-compliant with regards treatment consumption (<80%; although this did not apply to any participants in this study).

The group demographics are shown in Table 1. below. Two participants dropped out after their Day 1 assessment (for reasons unrelated to treatment) and were only included in the analysis of Day 1 data.

A small number of data points were either missing or omitted due to incorrect performance of tasks. Individual sample sizes for each task (n) are shown in the data tables in the Online Supplementary Materials.

#### Near-infrared Spectroscopy (NIRS) Cerebral Blood-Flow Participants

A sub-set of 41 participants (21 placebo, 20 ZA) took part in the cerebral blood-flow (CBF) assessment. Two participants in the placebo group were excluded from the assessment of the global effects of the treatments on resting CBF on Day 1/Day 56 and the acute CBF effects on Day 1 due to aberrant pre-dose baseline data due to a technical issue. The data from both participants was included in the assessment of hemodynamic responses on Day 1 and Day 56 (which used pre-task resting data as the baseline).

The study received ethical approval from the Northumbria University Psychology department (within the faculty of Health and Life Sciences) staff ethics committee (Reference 7905) and was conducted according to the Declaration of Helsinki (1964). All participants gave their written informed consent prior to their inclusion in the study.

### 2.3. Treatments

Treatments comprised 4 dark-brown soft gel capsules/day containing either:

- Placebo (sunflower oil)
- 2.8 g *Zanthozylum armatum* DC. Medium-chain triglyceride (MCT) oil extract (corresponding to 80 mg *Z.armatum* DC. extract)

The medium-chain triglyceride (MCT) oil extract of *Zanthozylum armatum* DC. (SaraPEPP™ Nu) was provided by Mibelle Group Biochemistry, Mibelle AG, Switzerland. Each 2.8 g dose contained alkamides including 7.8 mg hydroxy α-sanshool and monoterpenes including 5.3 mg limonene, 3.6 mg linalool and 2.3 mg methyl cinnamate.

Participants consumed the first and last dose of their 56-day treatment regimen under supervision within the laboratory. Otherwise, they consumed their treatment twice daily (2 capsules in the morning, 2 capsules in the evening, together with a meal) at home. The average compliance (as assessed by treatment diary and returned pill count) was 98%.

A treatment guess at the end of the study showed that there was no significant difference between the accuracy of guessing in the two treatment groups.

### 2.4. Cognitive and Mood Measures

**Figure 2:**
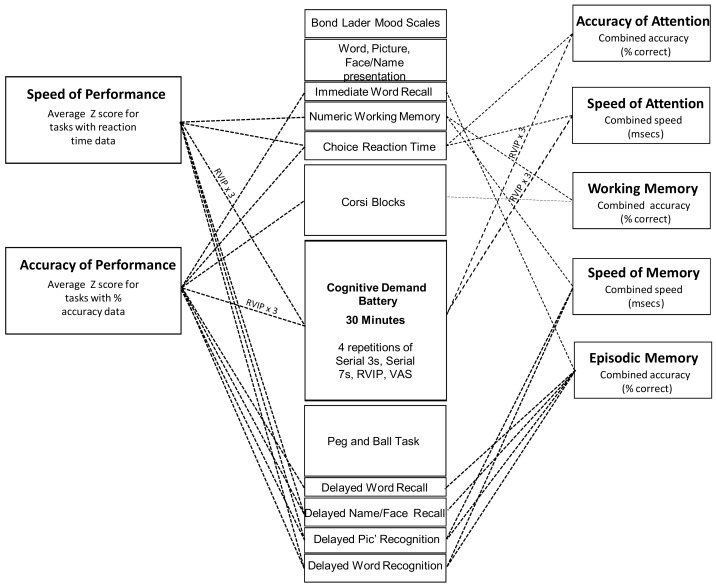
Cognitive assessments. The running order of tasks and their contribution to the cognitive factors (to the right) and global performance measures (to the left) derived from the overall battery. The selection of tasks took 60 min to complete (with the Cognitive Demand Battery comprising 30 min of this). The same assessment was completed at the pre-treatment baseline and at 1, 3 and 5 h post-dose during both Day 1 and Day 56.

All cognitive tasks and mood measures were delivered via the Computerized Mental Performance Assessment System (COMPASS; see: www.cognitivetesting.co.uk), a software platform for the presentation of classic and bespoke computerized cognitive tasks, with fully randomized parallel versions of each task delivered at each assessment for each individual. Tasks were presented on a laptop PC with responses made either via a four-button response box, with mouse and cursor or by the keyboard’s linear number pad. The tasks and other components of each assessment are described below in order of completion. The timelines of each assessment are shown in Figure 2. Given the exploratory nature of the study, a wide spectrum of classic, widely employed cognitive tasks, assessing attention, secondary and working memory and executive function, were employed. A similar selection of tasks has previously been shown to be sensitive to nutritional interventions [20,21,22,23,24].

#### 2.4.1. Bond-Lader Mood Scales

The Bond-Lader Mood Scales [25] have been utilised in numerous pharmacological, psychopharmacological and medical trials. These scales comprise a total of sixteen 100 mm lines anchored at either end by antonyms (e.g., ‘alert-drowsy,’ ‘calm-excited’). Participants indicate their current subjective position between the antonyms on the line. Outcomes comprise three factor analysis derived scores—‘Alertness,’ ‘Calmness’ and ‘Contentment.’

#### 2.4.2. Stimuli (Picture, Name/Face, Word) Presentation

Fifteen black-and-white photographic images of objects were presented sequentially on screen for the participant to remember at the rate of 1 every 3 s, with a stimulus duration of one second. Following this a set of twelve passport-style photographic images of people were presented sequentially to participants. A first and last name was assigned to each photograph and presented on the screen underneath the person’s face. Stimulus duration was one second, with a 3-s inter-stimulus duration. Finally, a unique set of fifteen words was presented. Words were selected at random from a large bank of words derived from the Medical Research Council Psycholinguistic Database [26] and matched for word length, frequency, familiarity and concreteness. Stimulus duration was one second, as was the inter-stimulus duration.

#### 2.4.3. Immediate Word Recall

The participant was allowed 60 s to write down as many of the words as possible. The task was scored for number correct and errors.

#### 2.4.4. Numeric Working Memory

Five random digits from 1–9 were presented sequentially for the participant to hold in memory. This was followed by a series of 30 probe digits (15 targets and 15 distractors) for each of which the participant indicated whether or not it had been in the original series by a simple key press. The task consisted of 3 separate trials. Accuracy (% correct) and mean reaction time (ms) were recorded.

#### 2.4.5. Corsi Blocks Task

In this task, nine identical blue squares appeared on screen in non-overlapping random positions. A set number of blocks changed color from blue to red in a randomly generated sequence. The cursor was locked in position until the entire sequence had been presented, at which point the participants were instructed to repeat the sequence by clicking on the blocks using the mouse and cursor. The task was repeated five times at each level of difficulty. The sequence span increased from 4 upwards, until the participant could no longer correctly recall the sequence, resulting in a span measure of nonverbal working memory, calculated by averaging the level of the last five correctly completed trials.

#### 2.4.6. Choice Reaction Time (CRT)

The Choice Reaction Task (CRT) task required participants to indicate, by pressing the ‘left’ or ‘right’ response box button, the direction of the arrow presented on the computer screen. Fifty stimuli (arrows) were presented, with a randomly varying delay of 1 to 3 s between stimuli. The task took approximately 2 min to complete, depending on participant reaction time. The task wass scored for % correct responses and reaction time (ms).

#### 2.4.7. Cognitive Demand Battery 

Multiple completions of this 10 min battery of tasks reliably increases self-ratings of mental fatigue and it has been shown to be sensitive to many natural interventions [19,27,28,29,30]. Two minutes each of Serial 3s and 7s subtractions is first completed and followed immediately by 5 min of Rapid Visual Information Processing. Mental fatigue is self-rated after each completion of the three tasks. In the current study the tasks were repeated 3 times, giving a total of 30 min of continuous demanding cognitive task performance.

Serial 3s and 7s subtractions—At the start of the 2 min task a standard instruction screen informs the participant to count backwards in 3s or 7s as quickly and accurately as possible, using the keyboards linear number keys to enter each response. Participants were instructed verbally at the outset that if they were to make a mistake they should carry on subtracting from the new incorrect number with subsequent responses scored as correct in relation to the new number. To begin, a random starting number between 800 and 999 was presented on the computer screen, which was cleared by the entry of the first response. Each three-digit response was represented on screen by asterisks and pressing the enter key signalled the end of each response and cleared the three asterisks from the screen. Outcomes were the total number of correct subtractions and the number of incorrect responses.

Rapid Visual Information Processing (RVIP) task—The RVIP task required the participant to monitor a continuous series of single digits for targets of three consecutive odd or even numbers. The white digits are presented on the black computer screen at the rate of 100 per minutes; with eight correct target strings in each minute presented in pseudo-random order. The participant responded to the detection of a target string by pressing the appropriate response button as quickly as possible. The RVIP was scored for the % target strings correctly detected, false alarms and the average reaction time (ms) for correct detections.

#### 2.4.8. Peg and Ball

Two configurations of wooden peg (×3) and ball (×3; blue, green and red) diagrams were displayed, on screen, with the top diagram denoting the ‘goal’ configuration of balls on pegs. Participants were required to rearrange the balls on the ‘starting’ configuration below this to match the ‘goal’ configuration. They were instructed to do this in the least number of moves possible. The task was scored for average thinking time (ms), average completion time (ms) and errors (total number of moves in excess of minimum required to complete all trials).

#### 2.4.9. Delayed Word Recall

Participants were informed, via a computerized instruction page, that they had 60 s to note down as many of the words from the list presented at the beginning of the task battery as they could remember. The task outcomes were the number of correctly recalled words and the number of incorrect words.

#### 2.4.10. Delayed Name to Face Recall

The target faces presented at the beginning of the battery were displayed on the screen one at a time. Below each face was a list of 4 forenames and a list of 4 surnames. Participants used the mouse to select the forename and surname that they believed were associated with each face as presented at the beginning of the assessment. The task outcomes comprised % accuracy for overall correct forenames and correct surnames and reaction time (ms).

#### 2.4.11. Delayed Picture and Word Recognition

Word and picture recognition were completed separately but both required participants to differentiate, by pressing ‘yes’ or ‘no’ on the response box, between the 15 target words and pictures presented at the beginning of the test battery and 15 randomly interspersed decoy words and pictures. The tasks took ~2 min to complete and were scored for % of correctly recognized words/pictures and reaction time (ms).

All of the computerized cognitive assessments were identical, with the exception of the presentation of randomly generated parallel versions of each task. The individual tasks making up the cognitive/mood assessment, alongside the timelines of each assessment are shown in Figure 2.

Figure 2 also shows the contribution of individual tasks to the measures that were derived by collapsing the data from individual tasks into cognitive domain factor scores representing the average speed (ms) of performance (Speed of Attention, Speed of Memory) or average (% correct) accuracy (Accuracy of Attention, Working Memory, Secondary Memory) of the attention tasks and working/episodic memory tasks within the battery. Two global performance measures were also calculated; ‘Speed of Performance’ and ‘Accuracy of Performance.’ These comprised averaged Z scores for speed and accuracy data respectively (for all tasks that collected such data). The composite measures have previously been shown to be sensitive to nutritional interventions ([23,31]). The task running order and contribution to cognitive factors and global measures are shown in Figure 2 below.

### 2.5. Cerebral Blood-Flow (via NIRS) Assessment

**Figure 3:**
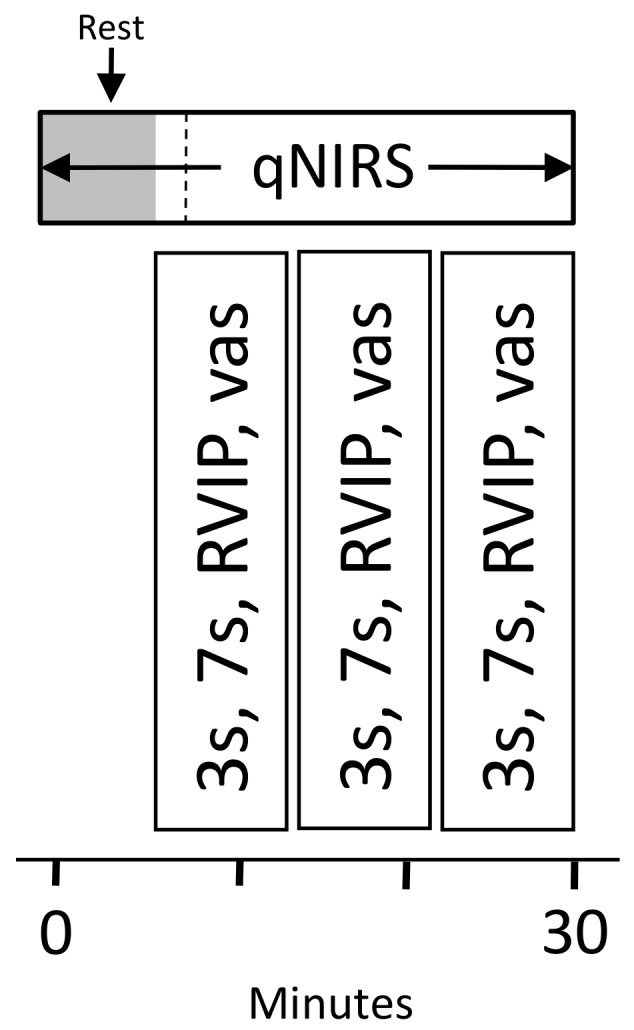
Near-infrared spectroscopy (NIRS) assessment timeline. Each NIRS assessment comprised a pre-dose resting baseline and a post-dose assessment taking place between the 60– and 180 min post-dose cognitive assessments, as shown here. The post-dose assessmentcomprised a 5 min resting period, followed by three repetitions of the Cognitive Demand Battery tasks (27 min), all of which have been shown to sensitively modulate CBF parameters in the frontal cortex previously.

Forty one of the participants took part in an additional NIRS assessment, with CBF parameters (oxygenated hemoglobin [oxy-Hb], deoxygenated hemoglobin [deoxy-Hb], total-hemoglobin [total-Hb—the sum of oxy-Hb and deoxy-Hb, a marker of overall CBF] and % oxygen saturation [% oxy]) measured in the frontal cortex using NIRS at rest and during cognitive task performance. Each assessment comprised a 5 min pre-dose resting measurement and a post-dose assessment commencing either at 120 or 150 min post-dose. The post-dose assessment comprised a 5 min resting measurement followed by 27 min (3 repetitions) of performing the Cognitive Demand Battery tasks (Serial 3s – 2 min, Serial 7s – 2 min, RVIP—5 min) all of which activate the frontal cortex. The NIRS assessment is shown in Figure 3.

#### Near-Infrared Spectroscopy (NIRS)

Cerebral blood-flow (CBF) parameters and hemodynamic responses during task performance were monitored using a quantitative NIRS system (OxiplexTS Frequency-Domain Near-Infrared Tissue Oximeter; ISS, Inc., Champaign, IL, USA). NIRS provides a proxy measure of CBF parameters and has been shown to sensitively measure resting CBF and hemodynamic responses in humans during differing levels of brain activation [32]. The system employed here gives absolute measurements of absorption of near-infrared light emitted at two distinct wavelengths. This allows the quantification of oxy-Hb and deoxy-Hb. These values are then used to determine total-Hb (oxy-Hb + deoxy-Hb) and % oxy (oxy-Hb/total-Hb × 100). This system is ideal for quantifying acute changes in hemodynamic responses over an extended period (i.e., with intermittent testing throughout one visit) and in a chronic context (here comparing CBF parameters between Day 1 and Day 56).

Light was emitted at 691 and 830 nm by optical fibers glued in pairs to four prisms (eight fibers in total) that were separated from the collector bundle, also glued to a prism, by 2.0-, 2.5-, 3.0- or 3.5 cm. Each of the emitter and collector bundle prisms were embedded into a flexible polyurethane resin to form a sensor with the overall dimensions of 7.6 cm × 2.5 cm × 0.3 cm. Identical sensors were attached to either side of the forehead of participants with medical tape and secured in place with a self-adhering bandage. The sensors were positioned so that the bottom edge was level with the top of the participants’ eyebrows and the middle edge touching at the midline of the forehead. Data were collected at a rate of 5 Hz.

### 2.6. Procedure

Testing took place in a suite of testing facilities with participants visually isolated from each other. Participants attended the laboratory on 3 separate occasions; an introductory visit between 1 and 14 days before the first day of treatment and two testing days (Day 1 and Day 56 of treatment).

The Introductory visit to the laboratory comprised—briefing on requirements of the study, obtaining of informed consent, health screening, completion of the Caffeine Consumption Questionnaire (CCQ) and State-Trait Anxiety Inventory (STAI) trait subscale, training on the cognitive and mood measures and collection of demographic data.

**Figure 4:**
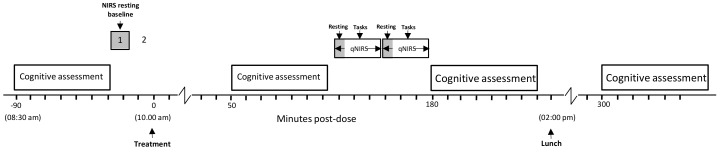
Testing session timeline on Day 1 and Day 56. Cognitive assessments were completed at 8:30 am (pre-dose) and 60-, 180- and 300 min post-dose. Cerebral blood flow measures were taken from a subset of participants at baseline and then again at post-dose between the 60- and 180 min cognitive assessments. A standardized lunch was provided at ~2:00 pm.

For the two subsequent laboratory-based testing sessions (Day 1, Day 56) participants attended the laboratory at 8:30 am having consumed a standardized breakfast of cereal and toast at home no later than an hour before arrival. They had refrained from alcohol for 24 h and caffeine for 18 h before each visit. On arrival on each day, participants completed the STAI state subscale and the computerized cognitive assessment (as per Figure 2). The participants who were taking part in the CBF study (maximum 2 participants per day) were then equipped with the NIRS for a 5-min resting measurement of CBF parameters. Thirty minutes after the initial cognitive assessment participants consumed their treatment for that day. Three further cognitive assessments, identical to the pre-dose assessment commenced at 1 h, 3 h and 5 h post-dose, with the CBF subset of participants undergoing their post-dose NIRS assessment between the 1 h and 3 h cognitive assessments (i.e., commencing at either 120 or 150 min post-dose). Participants were given a standardized lunch at approximately 2:00 pm. Testing was identical on Day 56 with the exception that—following the completion of Day 1 testing, participants were provided with the treatment they would be consuming between study days and a treatment diary; and following the completion of testing on Day 56, participants were asked to guess which treatment they had consumed and they returned their remaining treatment and diary for analysis of compliance and adverse events. The testing day timeline is shown in Figure 4.

### 2.7. Analysis

#### 2.7.1. Cognitive and Mood Outcomes

The data from the individual tasks was collapsed into five separate cognitive factor scores (accuracy measures = average % correct: speed measures = average reaction time in ms) and two global performance scores (average of accuracy or speed Z scores) as shown in Figure 2. The outcomes from the individual tasks shown in Figure 2 were also analyzed as secondary outcomes.

The 3 repetitions (per assessment) of the Cognitive Demand Battery also returned the outcomes described above for the mental fatigue visual analogue scale, RVIP task, Serial 3sand Serial 7s.

Acute (Day 1) effects—In order to explore the acute effects of ZA, data from the Day 1 post-dose assessments at 1 h, 3 h and 5 h were baseline adjusted with regards the pre-dose baseline data collected on Day 1 and entered into a two way (treatment [placebo, ZA] × assessment [1 h, 3 h, 5 h]) ANOVA. Analyses of the Cognitive Demand Battery outcomes were by three-way (treatment [placebo, ZA] × assessment [1, 3 h, 5 h] × repetition [1, 2, 3]) ANOVA.

Chronic (Day 56) effects—In order to explore the chronic effects of ZA after 56 days administration, data from the pre-dose and 1 h, 3 h and 5 h post-dose assessments on Day 56 were baseline adjusted with respect to the Day 1 pre-dose baseline data and analyzed via two-way (treatment [placebo, ZA] × assessment [pre-dose, 1 h, 3 h, 5 h]) ANOVA to compare performance between treatments. Analyses of the Cognitive Demand Battery outcomes were by three-way (treatment [placebo, ZA] × assessment [1 h, 3 h, 5 h] × repetition [1, 2, 3]) ANOVA.

For both the acute and chronic analyses, planned comparisons between data from each condition during each assessment were also conducted using *t*-tests, utilizing Mean Squares Error (MSE) from the ANOVA. These comparisons were Bonferroni adjusted. Only those planned comparisons relating to an outcome that generated a significant ANOVA result are reported. 

Day 1 baseline differences between placebo and ZA were explored using t-tests for those measures that had a single repetition and with reference to the main treatment effect of two-way ANOVAs (repetition [3] × treatment [placebo/ZA]) for those measures (Cognitive Demand Battery outcomes) that had three repetitions.

#### 2.7.2. Frontal Cortex Cerebral Blood Flow analysis

There were three separate analyses of data for these CBF parameters:

Global CBF effects on Day 1 and Day 56—The effects of ZA on global CBF parameters in the absence of task related brain activation (i.e., resting CBF) were analysed with unadjusted averaged data from the pre and post-dose resting assessments on Day 1 and Day 56. The analysis was by three-way ANOVA (pre/post dose × Day 1/56 × treatment) of averaged data from each 5 min rest period. Bonferroni adjusted post-hoc comparisons were then carried out between ZA and placebo as appropriate. This analysis also provided a test of baseline differences between groups on Day 1 and at rest (post-dose) on Day 1/Day 56.

Acute (Day 1) effects during brain activation—Post-dose data were averaged across 14 epochs (rest: 2 × 2.5 min, Serial 3s subtractions: 3 × 2 min, Serial 7s subtractions: 3 × 2 min, RVIP: 6 × 2.5 min: See Figures 9 and 10). The acute effects of a single dose of ZA on CBF parameters on Day 1 were measured as the change in CBF parameters during each 2 or 2.5 min epoch of the 32-min post-dose assessment, baseline adjusted to the pre-dose resting baseline. The analysis of change from baseline data was by two-way ANOVA (treatment [ZA/placebo] × epoch [14 × 2/2.5 min epochs). Bonferroni adjusted planned comparisons were then carried out between ZA and placebo during each epoch of the Day 1 post-dose assessment. Only those planned comparisons associated with a significant treatment related effect (multivariate or between-subjects) are reported below.

Hemodynamic responses on Day 1 and Day 56—In the absence of any effect on global blood-flow on Day 1/56 (see results below) the effects of ZA on hemodynamic responses (the CBF response to local brain activation; i.e., how much blood flow and oxygenation change in response to increased activity) were measured as changes in CBF parameters during each 2/2.5 min epoch of active task performance and baseline adjusted to the respective 5 min resting period immediately before task performance. The analysis of change from resting baseline data was performed by three-way ANOVA (treatment [ZA/placebo] × day [1/56] × epoch [12 × 2/2.5 min epochs]). Bonferroni adjusted planned comparisons were then carried out as described above.

## 3. Results

### 3.1. Cognitive Function and Mood

#### 3.1.1. Baseline Differences

There was a significant difference between groups in terms of self-rated mental fatigue during the Cognitive Demand Battery, with participants in the ZA group rating themselves as more mentally fatigued than placebo [F(1, 79) = 8.10, *p* = 0.006] during the Day 1 pre-dose assessment. Participants in the ZA group also made significantly more false alarms on the RVIP task during the Day 1 pre-dose assessment compared to placebo [F(1, 79) = 7.34, *p* = 0.008].

There were no other significant Day 1 baseline differences on any demographic, cognitive, mood or CBF measure.

#### 3.1.2. Acute Effects of ZA after a Single Dose (Day 1)

**Figure 5:**
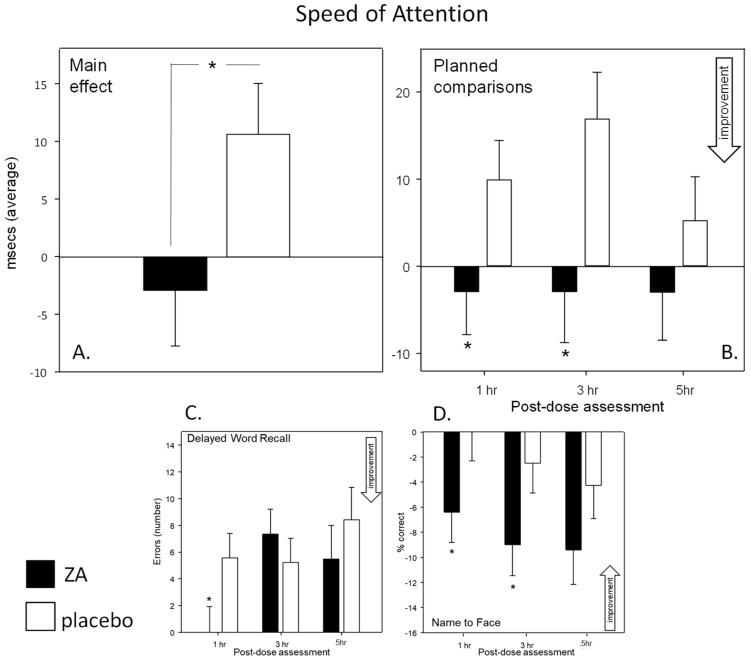
Effects on Speed of Attention. Top: Acute (Day 1) effects of a single dose of ZA on the Speed of Attention factor showing the main effect (panel **A**) and the planned comparisons (panel **B**) conducted on data from each post-dose assessment (i.e., 1-, 3- and 5-h post-dose). Bottom: acute effects of ZA on the individual cognitive tasks, Delayed Word Recall errors (panel **C**) and Name to Face recall accuracy (panel **D**). Data (mean and SEM) is change from pre-dose baseline. * = *p* < 0.05. *n* = 42/35 (ZA/placebo) for panel A and B and 42/39 for panel **C** and **D**.

Cognitive factors and global speed and accuracy outcomes—Consumption of a single dose of ZA lead to a significant improvement on the Speed of Attention factor (main effect—[F(1, 75) = 4.42, *p* = 0.039]). Reference to planned comparisons of data from each assessment showed that the speed of attention task performance was enhanced at both 1 h [*t*(150) = 3.17, *p* = 0.005] and 3 h [*t*(150) = 4.89, *p* < 0.001] post-dose. See Figure 5 panel A/B. In support of this there was a trend towards increased speed across the global Speed of Performance factor [F(1, 79) = 2.91, *p* = 0.092].

In terms of single tasks, there was a significant treatment × assessment interaction with regards the number of errors on the Delayed Word Recall task [F(2, 158) = 3.36, *p* = 0.037] with participants generating significantly fewer errors following ZA during the 1 h post-dose assessment [*t*(158) = 2.56, *p* = 0.011]. In contrast, participants performed the Name to Face task less accurately in terms of % correct following ZA [F(1, 79) = 4.35, *p* = 0.04] with this effect evident at 1 h [*t*(158) = 2.51, *p* = 0.039] and 3 h [*t*(158) = 2.55, *p* = 0.035]. See Figure 5 panel C/D. There were also significant treatment × assessment interactions for the Bond-Lader content score and Peg and Ball errors but planned comparisons showed that there was no significant difference between treatments during any of the three assessments.

**Figure 6:**
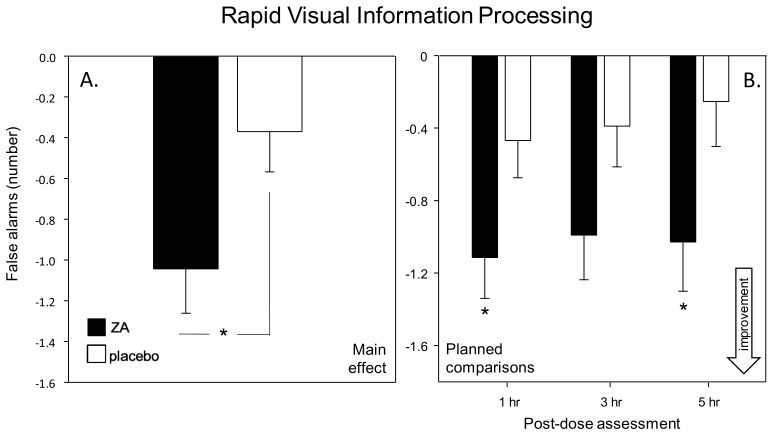
Effects on the Rapid Visual Information Processing (RVIP) task. Acute (Day 1) effects of a single dose of ZA on the RVIP task from the Cognitive Demand Battery outcomes. Panel **A** shows the main effect and panel B shows the planned comparisons conducted using data from each post-dose assessment (i.e., 1-, 3- and 5-h post-dose). Data (mean and SEM) are change from pre-dose baseline. * = *p* < 0.05 significant difference to placebo at that time point. *n* = 42/35 (ZA/placebo).

Cognitive Demand Battery—The accuracy of performance on the RVIP task was significantly improved following ZA in terms of reduced false alarms (main effect: [F(1, 75) = 5.25, *p* =0.025]). See Figure 6. Reference to the planned comparisons showed that this effect was significant during the 1 h [*t*(300) = 2.43, *p* = 0.046] and 5 h [*t*(300) = 2.95, *p* = 0.011] post-dose assessments. It is notable that there was a strong trend towards improved speed of performance on the RVIP [F(1, 75) = 3.80, *p* = 0.055] confirming that the increased accuracy was not due to a speed accuracy trade-off.

#### 3.1.3. Chronic Effects of ZA after 56 Days Treatment

**Figure 7:**
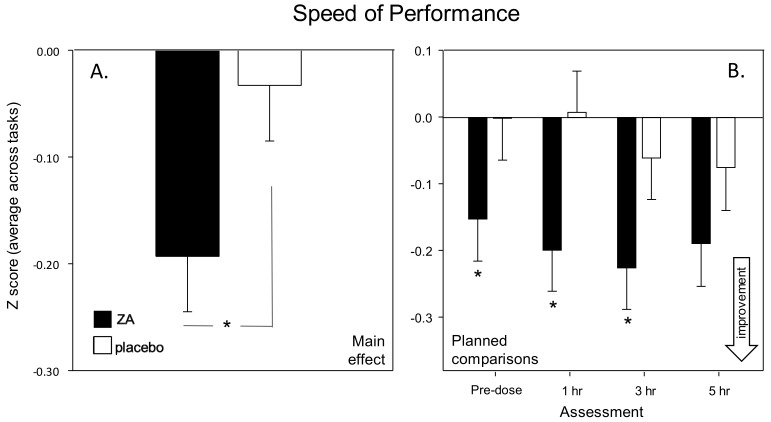
Effects on Speed of Performance. Chronic effects of 56-days administration of ZA on the global Speed of Performance measure (calculated as the average reaction time Z score for the 9 timed tasks). Panel **A** shows the main effect and panel B shows the planned comparisons conducted using data from each post-dose assessment (i.e., 1-, 3- and 5-h post-dose). Data (mean and SEM) are change from pre-dose baseline. * = *p* < 0.05 significant difference to placebo at that time point. *n* = 39/39 (ZA/placebo).

Cognitive factors and global speed and accuracy outcomes—There was a significant beneficial effect of ZA treatment on the global Speed of Performance measure [F(1, 76) = 4.28, *p* = 0.042]. See Figure 7. Planned comparisons of data from each assessment showed that participants performed significantly faster across the timed tasks during the pre-treatment [*t*(228) = 2.60, *p* = 0.02], 1 h [*t*(228) = 3.70, *p* < 0.001] and 3 h [*t*(228) = 2.92, *p* = 0.012] post-dose assessments. There were no significant effects of ZA with regards the other cognitive factor scores. However, it is noteworthy that there was a trend towards improved Speed of Attention across assessments on Day 56 [F(1,71) = 3.17, *p* = 0.079].

With respect to individual tasks, ZA resulted in an interaction between treatment and assessment for the speed of performing the Peg and Ball task [F(3, 222) = 4.71, *p* = 0.003]. Reference to the planned comparisons showed that the speed of performing the task was slower during the pre-dose assessment [*t*(222) = 2.91, *p* = 0.016] but numerically faster during later assessments.

**Figure 8:**
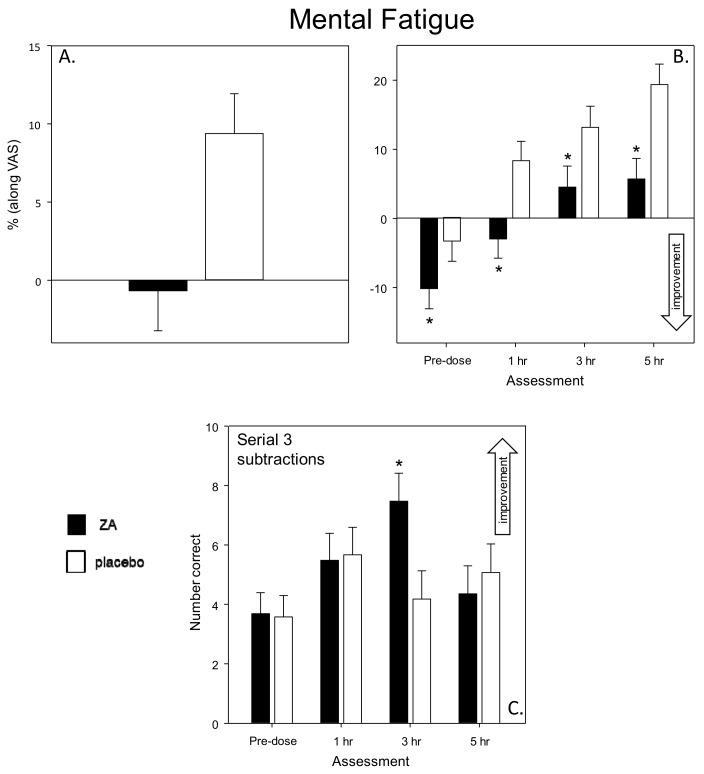
Chronic effects of ZA on Mental Fatigue and Serial 3s subtraction performance. Panel A shows the main effect on mental fatigue and panel B shows the planned comparisons conducted on data from each post-dose assessment (i.e., 1-, 3- and 5-h post-dose). The bottom panel (C) depicts the chronic effects of ZA on the Serial 3s subtraction task. Data (mean and SEM) are change from pre-dose baseline. * = *p* < 0.05 significant difference to placebo. *n* = 39/39 (ZA/placebo).

Cognitive Demand Battery—ZA resulted in reduced ratings of mental fatigue during all of the assessments on Day 56 (main effect: [F(1,76) = 8.01, *p* = 0.006] and a treatment × assessment interaction [F(2, 465) = 4.09, *p* = 0.019]. Planned comparisons showed that, following ZA, mental fatigue was significantly lower during the pre-dose assessment [t(465) = 4.49, *p* < 0.001] and at 1 h [*t*(465) = 7.70, *p* < 0.001], 3 h [*t*(465) = 5.80, *p* < 0.001] and 5 h [*t*(465) = 9.20, *p* < 0.001] post-dose. There was also a treatment × assessment interaction with regards the number of correct Serial 3s subtractions [F(3, 450) = 6.42, *p* < 0.001], with planned comparisons showing that this was a consequence of a significant improvement following ZA during the 3 h post-dose assessment [*t*(450) = 3.09, *p* = 0.008]. See Figure 8.

Whilst not reaching significance, it is noteworthy that, in light of the reduced hemodynamic responses seen during the RVIP task in the concomitant NIRS study (see below), there were trends towards improved performance on this task in terms of reduced false alarms [F(1, 71) = 3.24, *p* = 0.076] and faster performance [F(1, 71) = 3.74, *p* = 0.057]. This confirms that the hemodynamic changes in the NIRS assessment described below were not associated with any cognitive decrements.

Data (means, SEMs and ANOVA F and *p* values) for the cognitive function analyses are presented in online supplementary Table S1 (factors and global measures), Tables S2 and S3 (individual task outcomes), Tables S5 and S6 (Cognitive Demand Battery). There was no effect of treatment on ratings of anxiety as assessed by the STAI Inventory or on the three factors derived from the Bond-Lader scales.

Data (means, SEMs and ANOVA F and *p* values) for the mood measures are presented in online supplementary Table S4 (Bond-Lader Mood Scales) and Table S7 (STAI).

### 3.2. NIRS-CBF

#### 3.2.1. Global Changes in Resting CBF Parameters

There was no significant effect of ZA on gross levels of oxy-Hb, deoxy-Hb or their sum (total-Hb) or ratio (% O_2_) measured during the pre-dose and post-dose resting periods on Day 1 and Day 56.

#### 3.2.2. Acute Effects on Day 1

**Figure 9:**
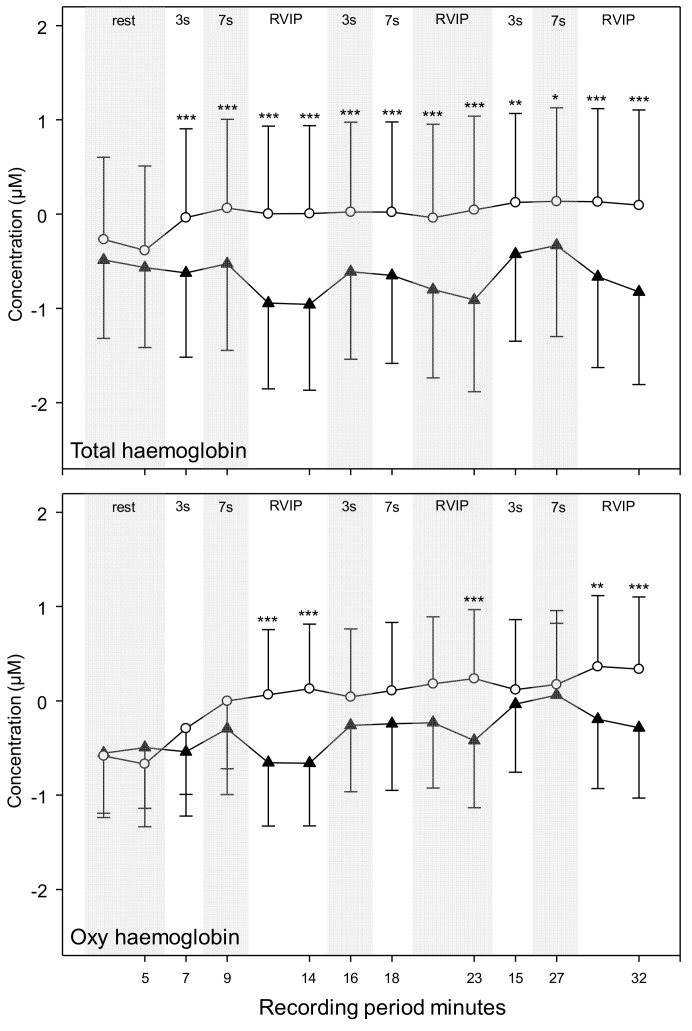
Acute effects on cerebral blood flow (CBF) parameters after the first dose of ZA. The figures show pre-dose resting baseline adjusted data (µM; mean + SEM) where placebo is compared to ZA at each 2/2.5 min epoch of pre-task rest and task performance during Serial 3s, Serial 7s and the Rapid Visual Information Processing tasks. Asterisks indicate significance on the Bonferroni adjusted planned comparisons conducted on data from each epoch. * = *p* < 0.05, ** = *p* < 0.01, *** = *p* < 0.001. *n* = 20/21 (ZA/placebo).

Analysis of change from pre-dose baseline data demonstrated that ZA resulted in significantly reduced oxy-Hb and total-Hb (i.e., CBF) during task performance (Total-Hb (multivariate) [F(13, 25) = 2.85, *p* = 0.012]−oxy-Hb [F(13, 481) = 1.95, *p* = 0.025]). There was no significant effect during the post-dose rest period. Planned comparisons during each epoch are presented in Figure 9. Reference to the figures shows that the effect is predominantly driven by a decreased hemodynamic response in terms of oxy-Hb, with this effect only significant during the RVIP attention task. There was no significant effect on deoxy-Hb or % O_2_.

#### 3.2.3. Acute (Day 1) and Chronic (Day 56) Effects on Hemodynamic Responses to Brain Activation

**Figure 10:**
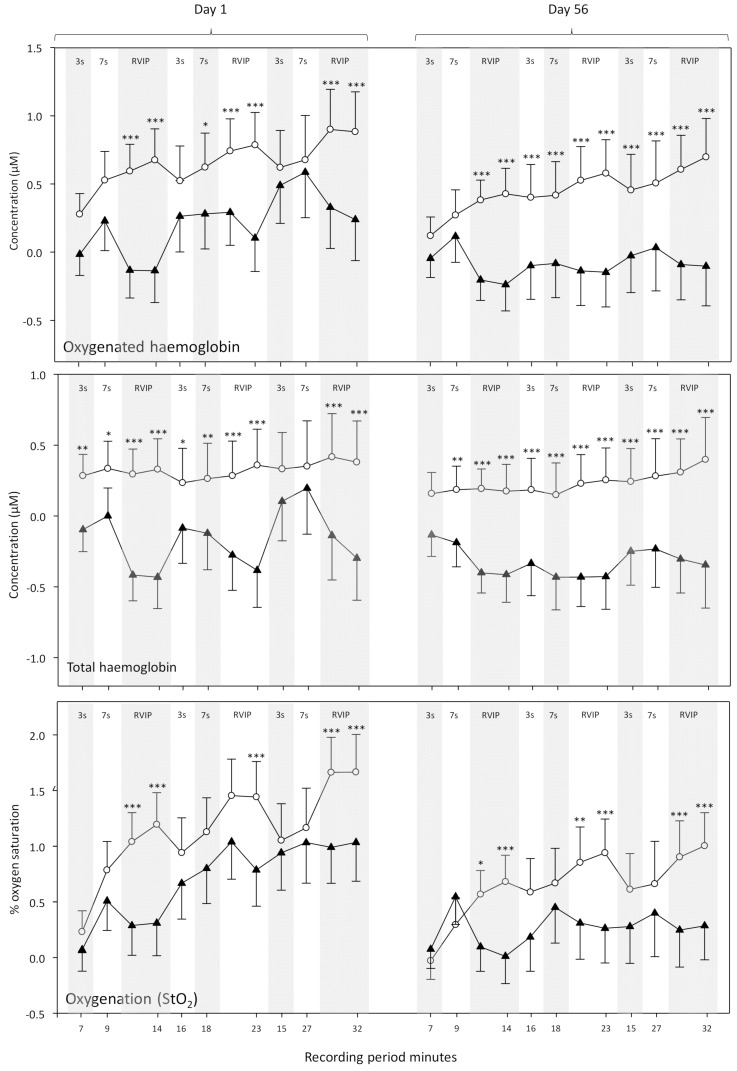
Effects of ZA on hemodynamic responses to brain activation (task performance) on Day 1 and Day 56 of treatment. The figures show data (mean + SEM) from each epoch of task performance (Serial 3s, Serial 7s and the Rapid Visual Information Processing tasks) (i.e., 7–32 min), baseline adjusted to the 5 min rest period immediately before the tasks commenced. Asterisks indicate significance on the Bonferroni adjusted planned comparisons; where placebo is compared to ZA at each epoch. * = *p* < 0.05, ** = *p* < 0.01, *** = *p* < 0.001. *n* = 20/21 (ZA/placebo).

Whilst consumption of ZA had no effect on global CBF parameter at rest (see above) it did result in significant modulation of the hemodynamic response to brain activation during task performance on both Day 1 and Day 56 (treatment × epoch interactions—oxy-Hb [F(11, 429) = 2.21. *p* = 0.013], % O_2_ [F(11, 429) = 2.38, *p* = 0.007]—main effect, total-Hb [F(1, 39) = 4.43, *p* = 0.042]). The pattern was for a global reduction in CBF driven by the reduction in oxygenated hemoglobin, which itself was most pronounced during the RVIP attention task. Oxygen saturation (% O_2_); reflecting the ratio of oxy-Hb and deoxy-Hb, was also significantly decreased but only during the RVIP task. Planned comparisons during each epoch are presented in Figure 10.

Data (means, SEMs—*t* and *p* values at each epoch) for the NIRS CBF-parameter analyses are presented in online supplementary Table S8 (acute effects on Day 1), Table S9 (pre/post-treatment resting CBF on Day 1/56) and Table S10 (acute and chronic hemodynamic response).

#### 3.2.4. Cognitive Performance

There were no significant acute or chronic treatment related changes in the performance of tasks (Serial 3s, Serial 7s, RVIP), during the NIRS assessment. Data (means and SEM) for the Cognitive Demand Battery tasks during the NIRS assessment are presented in supplementary Tables S11 and S12.

## 4. Discussion

The results demonstrate that a single dose and 56-days administration of *Zanthoxylum armatum* DC. MCT oil extract (ZA; product name SaraPEPP™ Nu) lead to significant improvements in cognitive function and modulation of cerebral blood flow (CBF) parameters.

With regards cognitive function, the most striking finding was an increased speed of task performance. This was seen in terms of significantly faster attention task performance (Speed of Attention) on Day 1 and significantly faster performance across all tasks (Speed of Performance) on Day 56. These findings were also supported by corresponding trends towards faster task performance on both days (Speed of Performance on Day 1 and Speed of Attention on Day 56). Improvements were also seen within the Cognitive Demand Battery outcomes. Firstly, improved accuracy of Rapid Visual Information Processing (RVIP) performance, in terms of false alarms after a single dose (Day 1), was observed. Reduced ratings of Mental Fatigue across all assessments and increased correct Serial 3 s were also evident during the 3 h post-dose assessment, on Day 56. Of note, the beneficial effects of ZA on Speed of Performance and Mental Fatigue on Day 56 were evident both before and, to a greater extent, after the consumption of the day’s treatments, suggesting that the beneficial effects of ZA, at least in part, were related to chronic consumption.

The predominant cognitive effects were a general increase in speed of processing, rather than domain specific changes and these might be expected to correlate with a similar general change in CBF during task performance. With reference to the NIRS results, whilst ZA had no effect on gross, resting CBF following either a single dose or 56-days administration, it did result in modulation of several hemodynamic parameters during the performance of tasks that activate the pre-frontal cortex; both following a single dose and following 56-days administration. Broadly, the pattern of results was for reduced hemodynamic responses during task performance across both days. For the analysis combining data from Day 1 and Day 56 this was seen in terms of reduced oxygenated hemoglobin, reduced total hemoglobin (a proxy for overall CBF) and reduced % oxygenation, with these effects seen predominantly during the RVIP attention task. A further analysis of CBF data (task × treatment × day) confirmed that the significant effects seen here were task dependent and primarily seen during the RVIP task. It is notable that performance of the RVIP task has previously been shown to activate the prefrontal cortex [33,34]. Interestingly, the ANOVAs here did not show any interactions between visit and treatment; suggesting that the hemodynamic effects during task performance were similar after acute and chronic supplementation. However, it might be noteworthy that the pattern of modulation after 56-days administration could be described descriptively as being stronger, on the basis of the number of epochs that saw a significant difference between treatments.

One important point here is that the reduced hemodynamic response, particularly during the RVIP focused attention task, was not associated with poorer cognitive function. Indeed, as well as the significant improvements in the speed of performing the attention tasks on Day 1 and all of the timed tasks on Day 56, performance on the RVIP task for the full cohort was more accurate on Day 1 with trends towards increased accuracy and faster speed on this task on Day 56. Taken together, these findings strongly suggest that the pattern of hemodynamic effects seen here reflect improved physiological brain function. It would, of course, be interesting to explore the nature and spatial properties of the brain function effects seen here with alternative imaging techniques. NIRS only provides proxy measures of CBF and these can be conflated with changes in cerebral blood volume and cerebral rates of oxygen metabolism. In common with NIRS, most other brain imaging techniques also interpret proxy markers. However, investigations of ZA’s effects on regional electrophysiological activity (e.g., via Electroencephalography (EEG)), gross cerebral blood flow parameters (e.g., via Trans-Cranial Doppler) or the spatial resolution of any brain function effects (via Functional Magnetic Resonance Imaging (FMRI) would be of interest in future trials. Interestingly, recently developed neuroimaging techniques, such as Event-related optical signal (EROS) offer a direct measure of neuronal cellular activity and might be useful in disentangling any changes in local neuronal activity and blood flow.

Interestingly, the CBF results in the current study bear a striking resemblance to those seen following the consumption of dietary nitrate [35]. In this previous study, which involved similar methodology as employed here, the reduction in CBF following a single dose of nitrate-rich beetroot juice was only seen during the RVIP task. There were no nitrate related effects on CBF during the serial subtraction tasks but improved performance on the Serial 3s subtraction task was seen nonetheless. In this earlier study it was noted that the RVIP task is a less cognitively demanding task and that the pattern of reductions in CBF represented an exaggeration of the typical response to reduced demand. The authors also attributed the findings to increased neural efficiency related to a nitrate related increase in the synthesis of the nitric oxide, with an attendant enhancement of cellular metabolic processes [35].

In terms of mechanisms underpinning the effects seen in the current study, previous research suggests that both the gastrointestinal effects of Daikenchuto, which contains hydroxy-alpha-sanshool from *Z. piperitum* [13] and the learning and memory effects of ZA in rodents [12] may be underpinned by increased cholinergic activity. Indeed, monoterpenes, which are represented in ZA extracts at significant levels, have been shown to have consistent cholinesterase inhibitory properties [36,37] and to exert beneficial cognitive effects [20]; including at doses as low as 25 µL [38]. A cholinergic mechanism certainly could be responsible for the cognitive effects seen here, although the pattern here on Day 56 was for a speeding of performance across cognitive domains; suggesting a general improvement in neural efficiency and processing speeds rather than the focused cognitive benefits to memory/attention that cholinergic agents are typically associated with. It is, however, unlikely that the interactions with cannabinoid CB1 and CB2 receptors attributed to some sanshools (specifically γ-sanshool) [14] played any role here, as preliminary screening demonstrated that the extract administered here did not exhibit any cannabinoid receptor binding activity (data not presented but available on request).

One alternative possibility then, is that the cellular ion channel mechanisms that drive the sensory tingling-numbness following topical/oral application of hydroxy α-sanshool; namely interactions with TRP and KCNK ion channels, may also be responsible for the cognitive and CBF effects seen here. In this respect it is notable that structurally similar alkamides have been shown to have good bioavailability and can readily cross the blood brain barrier [5]. Recent evidence also shows that the TRPV1 vanilloid receptors with which hydroxy α-sanshool interacts are widely distributed in the brain. Indeed, TRPV1 has a key role in the regulation of neuronal excitability and synaptic plasticity, including long-term potentiation, potentially mediated by glutamate release [17]. Similarly, several members of the KCNK two-pore domain potassium ion channel family that are inhibited by hydroxy α-sanshool, specifically TASK-1, TASK-3 and TRESK [4] are also expressed, often as TASK-1/TASK-3 heterodimers, throughout multiple brain regions, with particular concentration in the cerebellum, somatic motor-neurons and hypothalamus. These channels contribute to the excitability of distinct populations of neurons [18]. As an example, TASK-1 and TASK-3 channels contribute to the excitability of several types of hippocampal neurons, including CA1 pyramidal cell and inhibitory interneurons [39] and modulate the excitability of the key acetylcholine producing basal forebrain cholinergic neurons which drive elements of cortical arousal [40]. Clearly, interactions with neuronal mechanisms such as these might be expected to have a general, rather than cognitive-domain specific effect in terms of neural activation/efficiency and hemodynamic responses to brain activation.

## 5. Conclusions

In conclusion, a ZA extract rich in hydroxy α-sanshool resulted in global improvements in the speed of performing cognitive tasks, suggesting enhanced processing speed. ZA also resulted in a decreased hemodynamic response in the frontal cortex to cognitive task-mediated activation of this brain region. These effects can reasonably be interpreted as reflecting a ZA-related increase in neural efficiency, particularly during attention task performance. Whilst the exact mechanisms driving these effects are unclear at present, future research should explore the possibility that hydroxy α-sanshool is the active principal, potentially acting via interactions with neuronal ion channels.

## Figures and Tables

**Table 1:** Demographics of Zanthoxylum armatum DC. (ZA)and placebo groups. There were no significant differences between groups on any measure.

	Placebo	ZA
Number of Participants	42	41
Age at Enrolment (Years)	41.76	43.15
Sex	F30/M12	F33/M8
Years in Education	16.2	15.9
Portions of Fruit & Veg/day	4.37	4.42
Alcohol consumption daily (units)	0.84	0.72
Caffeine Consumption Score (mg)	149.90	179.67
Average BP Systolic (mm Hg)	116.92	116.88
Average BP Diastolic (mm Hg)	78.88	79.21
Average Heart Rate (Beats per min)	73.11	72.22
Weight (KG)	69.51	73.15
Height (cm)	168.51	167.47
BMI	24.47	25.92

## Supplementary Materials

The following are available online at https://www.mdpi.com/2072-6643/11/12/3022/s1, Table S1: Factors derived from the cognitive tasks and the global ‘Speed of Performance’ and ‘Accuracy of Performance’ outcomes (averaged Z scores) derived from the entire battery; Table S2: Acute Day 1 data for the individual cognitive task outcomes; Table S3: Chronic Day 56 data for the individual cognitive task outcomes; Table S4: Bond-Lader mood scale outcomes; Table S5: Acute Day 1 data for the Cognitive Demand Battery outcomes; Table S6: Chronic Day 56 data for the Cognitive Demand Battery outcomes; Table S7: State-Trait Anxiety Inventory; Table S8: Acute effects of ZA on cerebral blood-flow parameters (total haemoglobin, deoxygenated haemoglobin, oxygenated haemoglobin, % oxygen saturation) on Day 1; Table S9: Global effects of ZA on cerebral blood-flow parameters (total haemoglobin, deoxygenated haemoglobin, oxygenated haemoglobin, % oxygen saturation); Table S10: Acute and chronic effects of ZA on haemodynamic responses to task performance; Table S11: Data for the Cognitive Demand Battery outcomes during the qNIRS assessment on Day 1 (all mean plus SEM); Table S12: Data for the Cognitive Demand Battery outcomes during the qNIRS assessment on Day 56 (all mean plus SEM).

## Clinical Trials Number

NCT03673930.

## Abbreviations

Cerebral blood flow, CBF; *Zanthoxylum armatum*, ZA; Near-Infrared Spectroscopy, NIRS; oxygenated hemoglobin, oxy-Hb; deoxygenated hemoglobin, deoxy-Hb; total hemoglobin, total-Hb; oxygen saturation, % oxy; analysis of variance, ANOVA.
