# Supplementary material for: The Acute and Chronic Cognitive and Cerebral Blood-Flow Effects of Nepalese Pepper (Zanthoxylum armatum DC.) Extract—A Randomized, Double-Blind, Placebo-Controlled Study in Healthy Humans
Source: Nutrients. 2019 Dec 10;11(12):3022. doi: 10.3390/nu11123022 (PMC6950039; doi:10.3390/nu11123022)
Supplement: Supplementary file 1 [file nutrients-11-03022-s001.pdf]

Supplementary Online Materials - Tables S1 to S11

The acute and chronic cognitive and cerebral blood-flow effects of Nepalese Pepper (*Zanthoxylum armatum*) extract: a randomized, double-blind, placebo-controlled study in healthy humans

Table S1. Factors derived from the cognitive tasks and ***the global 'Speed of Performance' and 'Accuracy of Performance' outcomes*** *(averaged Z scores) derived from the entire battery. The top half of the table shows the data (mean plus SEM) related to the acute analysis of Day 1 data, and the bottom half of the table shows the data related to the chronic analysis of Day 56 data (plus Day 1 pre-dose baseline data). The ANOVA columns show the F scores and probabilities from the two-way ANOVA for that outcome: TxA = the treatment x assessment interaction term; T = the main effect of treatment term.*

DAY 1 (acute)

Measure			Baseline		Assessment						ANOVA		
		N	Day 1 pre-dose		1 hour		3 hour		6 hour			F	P
**Speed of**	pl	42	489.4	*8.24*	9.96	*4.47*	16.95	*5.31*	5.28	*4.99*	TxA	2.02	0.14
**Attention** (msec)	ZA	35	489.9	*6.84*	-2.95	*4.90*	-2.94	*5.82*	-3.01	*5.46*	T	4.43	0.04
**Accuracy of**	pl	42	77.12	*1.60*	-0.41	*0.63*	-1.32	*0.81*	-2.64	*0.89*	TxA	0.05	0.95
**Attention** (%)	ZA	35	76.59	*1.48*	-0.87	*0.68*	-2.11	*0.89*	-3.35	*0.98*	T	0.45	0.51
**Working**	pl	41	80.92	*0.92*	0.59	*1.11*	0.64	*0.98*	0.63	*1.12*	TxA	0.43	0.65
**Memory** (%)	ZA	38	79.48	*1.08*	-0.93	*1.15*	0.38	*1.02*	-0.68	*1.16*	T	0.63	0.43
**Speed of**	pl	41	911.8	*23.0*	-9.35	*11.0*	-23.3	*11.9*	-22.3	*13.6*	TxA	0.43	0.65
**Memory** (msec)	ZA	38	886.1	*20.5*	-19.7	*11.4*	-18.6	*12.3*	-30.7	*14.1*	T	0.11	0.74
**Episodic**	pl	42	60.24	*1.33*	-3.44	*1.03*	-4.20	*1.23*	-5.55	*1.13*	TxA	1.15	0.32
**Memory** (%)	ZA	39	59.48	*1.37*	-3.21	*1.07*	-6.36	*1.28*	-6.53	*1.18*	T	0.51	0.48
**Accuracy of**	pl	42	0.12	*0.08*	-0.12	*0.05*	-0.14	*0.06*	-0.24	*0.06*	TxA	1.63	0.20
**Performance (Z)**	ZA	39	0.04	*0.08*	-0.10	*0.05*	-0.24	*0.06*	-0.32	*0.06*	T	0.53	0.47
**Speed of**	pl	42	0.03	*0.09*	0.03	*0.05*	0.04	*0.05*	-0.08	*0.05*	TxA	0.12	0.89
**Performance (Z)**	ZA	39	0.13	*0.09*	-0.09	*0.05*	-0.09	*0.05*	-0.18	*0.06*	T	3.50	0.07

DAY 56 (chronic)

			Baseline		Assessment								ANOVA		
			Day 1 pre-dose		Pre-dose		1 hour		3 hour		6 hour			F	P
**Speed of**	pl	38	489.6	*8.89*	4.24	*7.12*	9.34	*6.34*	12.11	*5.97*	7.51	*5.98*	TxA	1.11	0.35
**Attention** (msec)	ZA	35	489.9	*6.84*	-6.96	*7.41*	-10.2	*6.61*	-6.11	*6.22*	-0.88	*6.23*	T	3.18	0.08
**Accuracy of**	pl	38	77.97	*1.68*	0.46	*0.92*	-0.40	*1.05*	-1.16	*1.03*	-2.31	*1.10*	TxA	0.77	0.51
**Attention** (%)	ZA	35	76.59	*1.48*	0.70	*0.96*	1.26	*1.09*	-0.28	*1.08*	-1.52	*1.14*	T	0.43	0.51
**Working**	pl	38	80.99	*0.97*	-0.23	*1.15*	-0.27	*1.19*	-0.80	*1.28*	-0.68	*1.25*	TxA	0.42	0.74
**Memory** (%)	ZA	38	79.48	*1.08*	1.14	*1.15*	0.78	*1.19*	1.57	*1.28*	0.65	*1.25*	T	0.98	0.33
**Speed of**	pl	38	914.0	*24.5*	5.47	*15.93*	-13.4	*15.6*	-36.5	*13.6*	-44.0	*16.5*	TxA	0.54	0.66
**Memory** (msec)	ZA	38	886.1	*20.5*	-24.9	*15.93*	-23.4	*15.6*	-53.0	*13.6*	-45.7	*16.5*	T	0.78	0.38
**Episodic**	pl	39	60.46	*1.37*	1.94	*1.06*	-2.00	*1.23*	-3.56	*1.08*	-4.09	*1.18*	TxA	0.42	0.74
**Memory** (%)	ZA	39	59.48	*1.37*	1.98	*1.06*	-3.14	*1.23*	-4.51	*1.08*	-5.72	*1.18*	T	0.49	0.49
**Accuracy of**	pl	39	0.15	*0.08*	0.11	*0.05*	-0.03	*0.06*	-0.11	*0.06*	-0.16	*0.07*	TxA	0.23	0.87
**Performance (Z)**	ZA	39	0.04	*0.08*	0.16	*0.05*	-0.01	*0.06*	-0.07	*0.06*	-0.17	*0.07*	T	0.13	0.71
**Speed of**	pl	39	0.02	*0.09*	-0.00	*0.06*	0.01	*0.06*	-0.06	*0.06*	-0.08	*0.06*	TxA	0.49	0.69
**Performance (Z)**	ZA	39	0.13	*0.09*	-0.15	*0.06*	-0.20	*0.06*	-0.23	*0.062*	-0.19	*0.06*	T	4.77	0.03

Table S2. Acute Day 1 data for the individual cognitive task outcomes. Showing pre-dose Day 1 raw baseline data and post-dose change from baseline data (all mean plus SEM). The ANOVA columns show the F scores and probabilities from the two-way ANOVA for that outcome: TxA = the treatment x assessment interaction term; T = the main effect of treatment term.

Task		N	Baseline		Post-dose assessment						ANOVA		
			Day 1 pre-dose		1 hour post-dose		3 hour post-dose		6 hours post-dose			F	P
**Immediate Word Recall** (% correct)	pl	42	42.14	*2.03*	-4.60	*1.99*	-4.05	*2.26*	-4.92	*1.85*	TxA	0.37	0.69
	ZA	39	38.21	*2.10*	-2.22	*2.06*	-4.27	*2.34*	-3.59	*1.92*	T	0.25	0.62
**Immediate Word recall** (errors)	pl	42	4.13	*0.82*	0.79	*0.99*	-0.79	*1.09*	2.70	*1.17*	TxA	2.87	0.06
	ZA	39	4.79	*1.12*	0.00	*1.02*	1.37	*1.13*	1.37	*1.22*	T	0.00	0.99
**Numeric Working Mem** (% correct)	pl	41	95.53	*0.64*	0.46	*0.87*	1.74	*0.69*	0.27	*0.81*	TxA	1.16	0.32
	ZA	38	95.70	*0.69*	0.47	*0.91*	-0.20	*0.71*	-0.47	*0.85*	T	1.05	0.31
**Numeric Working Mem RT** (msecs)	pl	41	880.7	*24.6*	-44.8	*15.9*	-36.4	*15.5*	-42.5	*15.7*	TxA	0.67	0.51
	ZA	38	847.8	*25.5*	-57.2	*16.5*	-63.3	*16.1*	-76.8	*16.3*	T	1.55	0.22
**Choice Reaction Time** (% correct)	pl	42	98.33	*0.39*	-0.10	*0.46*	0.05	*0.34*	-0.29	*0.30*	TxA	0.51	0.60
	ZA	39	97.69	*0.55*	0.21	*0.48*	0.00	*0.35*	-0.56	*0.31*	T	0.00	0.98
**Choice Reaction Time RT** (msecs)	pl	42	482.2	*13.0*	14.91	*7.53*	27.09	*9.67*	11.60	*8.87*	TxA	1.10	0.34
	ZA	39	463.1	*10.4*	1.01	*7.81*	6.62	*10.04*	6.31	*9.20*	T	1.42	0.24
**Corsi Span** (capacity score)	pl	42	5.98	*0.14*	0.07	*0.16*	-0.04	*0.16*	0.08	*0.19*	TxA	1.65	0.19
	ZA	39	5.62	*0.19*	-0.16	*0.17*	0.12	*0.17*	-0.04	*0.19*	T	0.09	0.77
**Peg and Ball think time** (msecs)	pl	41	3518	*237*	-300	*140*	-682	*139*	-659	*161*	TxA	1.01	0.37
	ZA	38	3662	*227*	-449	*145*	-663	*145*	-840	*167*	T	0.29	0.59
**Peg and Ball time** (msecs)	pl	41	9740	*417*	-640	*227*	-1490	*232*	-1408	*257*	TxA	2.60	0.08
	ZA	38	10247	*471*	-833	*235*	-1040	*241*	-1297	*267*	T	0.16	0.69
**Peg and Ball errors** (number)	pl	42	3.21	*0.44*	0.45	*0.68*	-1.24	*0.53*	-0.31	*0.63*	TxA	3.49	0.03
	ZA	38	3.47	*0.53*	-0.21	*0.71*	0.03	*0.56*	1.18	*0.66*	T	0.92	0.34
**Name to Face** (% correct)	pl	42	65.08	*2.56*	0.00	*2.30*	-2.48	*2.39*	-4.27	*2.64*	TxA	0.09	0.91
	ZA	39	65.60	*2.41*	-6.41	*2.39*	-8.97	*2.48*	-9.40	*2.74*	T	4.36	0.04
**Name to Face** (msecs)	pl	42	10661	*567*	-358	*706*	-316	*695*	-1115	*758*	TxA	0.50	0.61
	ZA	39	11614	*656*	-1253	*732*	-245	*722*	-1710	*786*	T	0.30	0.59
**Delayed word recall** (% correct)	pl	42	27.54	*2.18*	-10.9	*2.31*	-12.22	*2.38*	-15.24	*2.26*	TxA	2.63	0.11
	ZA	39	23.59	*2.21*	-4.96	*2.40*	-11.03	*2.47*	-9.15	*2.34*	T	1.36	0.26
**Delayed word recall** (errors)	pl	42	7.62	*1.34*	5.56	*1.84*	5.24	*1.80*	8.41	*2.42*	TxA	3.36	0.04
	ZA	39	7.52	*1.51*	0.00	*1.91*	7.35	*1.86*	5.47	*2.51*	T	0.80	0.37
**Word Recog'tion** (% correct)	pl	42	73.65	*1.51*	-1.75	*1.62*	-1.03	*1.68*	-2.38	*1.81*	TxA	3.03	0.05
	ZA	39	75.04	*1.71*	-0.34	*1.68*	-3.25	*1.74*	-6.15	*1.88*	T	0.52	0.47
**Word Recog'tion RT** (msecs)	pl	42	984.4	*36.1*	-15.1	*23.70*	-54.84	*23.38*	-32.49	*28.32*	TxA	0.18	0.83
	ZA	39	950.4	*32.9*	0.41	*24.60*	-34.50	*24.26*	-32.77	*29.39*	T	0.16	0.69
**Picture Recog'ion** (% correct)	pl	42	92.78	*1.21*	0.00	*1.17*	-1.19	*1.21*	-0.95	*1.24*	TxA	0.36	0.70
	ZA	39	94.96	*1.02*	-2.14	*1.21*	-4.27	*1.25*	-4.36	*1.29*	T	3.73	0.06
**Picture Recog'ion** (msecs)	pl	42	869.4	*21.1*	34.06	*22.77*	23.57	*23.80*	1.96	*18.90*	TxA	0.89	0.41
	ZA	39	869.6	*19.8*	9.28	*23.63*	40.48	*24.70*	15.42	*19.61*	T	0.01	0.94

Table S3. Chronic Day 56 data for the individual cognitive task outcomes. Showing pre-dose Day 1 raw baseline data and Day 56 change from baseline data (all means plus SEM). The ANOVA columns show the F scores and probabilities from the two-way ANOVA for that outcome: TxA = the treatment x assessment interaction term; T = the main effect of treatment term.

			**Baseline**		**Day 56 assessment**								**ANOVA**		
		N	***Day 1 Pre-dose***		***Pre-dose***		***1 hr post-dose***		***3 hr post-dose***		***6 hr post-dose***			**F**	**P**
**Immediate Word**	pl	39	42.65	*2.14*	1.11	*1.75*	1.03	*1.99*	-1.28	*2.01*	-2.56	*2.22*	TxA	0.37	0.78
**Recall** (% correct)	ZA	39	38.21	*2.10*	3.16	*2.27*	-0.26	*2.35*	-1.37	*1.88*	-2.39	*1.83*	T	0.01	0.92
**Immediate Word**	pl	39	4.10	*0.87*	0.51	*1.08*	-1.03	*1.08*	1.20	*1.27*	0.51	*1.02*	TxA	0.84	0.47
**recall** (errors)	ZA	39	4.79	*1.12*	-1.20	*1.29*	-0.51	*1.11*	-0.68	*0.88*	0.00	*1.12*	T	0.59	0.44
**Numeric Working**	pl	38	95.41	*0.68*	1.20	*0.68*	1.32	*0.60*	0.94	*0.79*	1.29	*0.82*	TxA	0.80	0.50
**Mem** (% correct)	ZA	38	95.70	*0.69*	1.40	*0.70*	0.29	*0.78*	0.82	*0.85*	0.53	*0.78*	T	0.22	0.64
**Numeric Working**	pl	38	872.8	*25.8*	-11.8	*19.87*	-42.8	*19.97*	-58.0	*19.63*	-49.7	*22.25*	TxA	0.81	0.49
**Mem RT** (msecs)	ZA	38	847.8	*25.5*	-31.1	*16.51*	-86.7	*17.37*	-76.5	*17.06*	-94.3	*15.54*	T	2.01	0.16
**Choice Reaction**	pl	39	98.41	*0.40*	0.00	*0.40*	-0.26	*0.36*	0.51	*0.42*	-0.05	*0.38*	TxA	0.68	0.56
**Time** (% correct)	ZA	39	97.69	*0.55*	0.10	*0.52*	0.00	*0.47*	0.31	*0.54*	0.51	*0.54*	T	0.10	0.75
**Choice Reaction**	pl	39	486.1	*13.8*	11.63	*12.34*	15.38	*12.48*	20.51	*11.85*	13.56	*10.94*	TxA	0.64	0.59
**Time RT** (msecs)	ZA	39	463.1	*10.4*	9.21	*12.78*	-0.06	*10.73*	5.69	*9.42*	11.08	*9.29*	T	0.40	0.53
**Corsi Span**	pl	39	6.00	*0.15*	-0.15	*0.21*	-0.18	*0.23*	-0.22	*0.26*	-0.22	*0.26*	TxA	0.42	0.74
(capacity score)	ZA	39	5.62	*0.19*	0.11	*0.17*	0.17	*0.19*	0.26	*0.19*	0.13	*0.16*	T	1.78	0.19
**Peg and Ball**	pl	38	3582	*235*	-624	*189*	-854	*186*	-857	*182*	-1122	*171*	TxA	1.58	0.20
**think time** (msecs)	ZA	38	3662	*227*	-383	*189*	-828	*186*	-998	*182*	-1057	*171*	T	0.04	0.84
**Peg and Ball time**	pl	38	9760	*439*	-1194	*324*	-1527	*344*	-1374	*371*	-1762	*388*	TxA	4.71	0.00
(msecs)	ZA	38	10247	*471*	-442	*324*	-1454	*344*	-1896	*371*	-2117	*388*	T	0.00	0.98
**Peg and Ball**	pl	39	3.33	*0.46*	-1.15	*0.53*	-0.33	*0.73*	0.67	*0.67*	-0.23	*0.63*	TxA	0.55	0.65
**errors** (number)	ZA	38	3.47	*0.53*	-0.74	*0.65*	-0.79	*0.65*	-0.03	*0.61*	-0.29	*0.74*	T	0.07	0.79
**Name to Face** (%	pl	39	65.28	*2.73*	0.32	*2.33*	-3.74	*2.20*	-4.91	*2.44*	-6.52	*2.24*	TxA	1.08	0.30
correct)	ZA	39	65.60	*2.41*	-1.82	*2.87*	-8.33	*3.28*	-8.01	*2.45*	-8.87	*2.74*	T	0.19	0.90
**Name to Face**	pl	39	10720	*598*	-1446	*561*	-146	*826*	-1038	*704*	-189	*760*	TxA	1.76	0.16
(msecs)	ZA	39	11614	*656*	-1034	*561*	-1755	*826*	-1940	*704*	-1845	*760*	T	1.38	0.24
**Delayed word**	pl	39	27.95	*2.28*	4.02	*2.02*	-8.89	*1.84*	-9.40	*2.61*	-9.91	*2.29*	TxA	0.96	0.41
**recall** (% correct)	ZA	39	23.59	*2.21*	6.41	*2.24*	-4.44	*2.34*	-10.26	*1.81*	-9.83	*2.07*	T	0.46	0.50
**Delayed word**	pl	39	7.35	*1.38*	0.51	*1.42*	2.22	*1.46*	3.59	*1.91*	5.98	*2.48*	TxA	0.37	0.77
**recall** (errors)	ZA	39	7.52	*1.51*	-1.03	*1.26*	2.91	*2.28*	4.44	*1.95*	4.96	*2.43*	T	0.01	0.91
**Word Recog'tion**	pl	39	73.25	*1.48*	3.50	*1.38*	1.54	*1.43*	-0.51	*1.80*	1.11	*1.60*	TxA	1.98	0.12
(% correct)	ZA	39	75.04	*1.71*	2.22	*1.48*	-0.34	*1.94*	0.51	*1.59*	-3.33	*2.05*	T	0.73	0.39
**Word Recog'tion**	pl	39	995.0	*38.4*	-43.5	*30.40*	-24.9	*25.39*	-75.00	*28.59*	-78.8	*32.58*	TxA	0.27	0.85
**RT** (msecs)	ZA	39	950.4	*32.9*	-40.9	*21.27*	7.03	*30.48*	-71.50	*25.29*	-58.8	*28.28*	T	0.21	0.65
**Picture Recog'ion**	pl	39	93.16	*1.26*	0.77	*1.26*	0.08	*1.32*	-1.71	*1.36*	-2.57	*1.26*	TxA	0.36	0.78
(% correct)	ZA	39	94.96	*1.02*	-0.09	*0.94*	-2.31	*1.26*	-3.42	*1.19*	-4.19	*1.35*	T	1.18	0.28
**Picture Recog'ion**	pl	39	872.9	*22.3*	67.51	*39.04*	27.32	*18.35*	27.56	*19.77*	-2.21	*21.41*	TxA	1.32	0.27
(msecs)	ZA	39	869.6	*19.8*	13.73	*24.75*	6.59	*21.62*	-11.94	*20.34*	21.32	*23.04*	T	0.94	0.33

Table S4. Bond-Lader mood scale outcomes. The top half of the table shows the data (mean plus SEM) related to the acute analysis of Day 1 data, and the bottom half of the table shows the data related to the chronic analysis of Day 56 data (plus Day 1 pre-dose baseline data). The ANOVA columns show the F scores and probabilities from the two-way ANOVA for that outcome: TxA = the treatment x assessment interaction term; T = the main effect of treatment term.

Day 1 (acute)

**Bond-Lader factor**			**Baseline** Day 1 pre-dose		**Post-dose Assessment** 1 hour		3 hour		6 hour		ANOVA	F	P
**Alert**	pl	42	68.42	*2.17*	-2.48	*1.54*	-9.65	*2.22*	-8.35	*2.21*	TxA	0.29	0.75
	ZA	39	67.25	*2.26*	-1.89	*1.60*	-9.60	*2.31*	-6.33	*2.30*	T	0.13	0.72
**Calm**	pl	42	71.61	*2.23*	-1.52	*1.72*	-2.61	*1.76*	-1.31	*1.81*	TxA	0.07	0.93
	ZA	39	67.62	*2.34*	-1.65	*1.78*	-2.99	*1.83*	-0.86	*1.87*	T	0.00	0.99
**Content**	pl	42	75.56	*1.92*	-1.95	*1.00*	-3.15	*1.53*	-3.80	*1.33*	TxA	3.58	0.03
	ZA	39	74.06	*2.42*	-0.44	*1.03*	-5.68	*1.59*	-2.05	*1.38*	T	0.02	0.88

Day 56 (chronic)

			**Baseline** Day 1 pre-dose		**Day 56 assessment** Pre-dose		1 hour		3 hour		6 hour				
**Alert**	pl	39	67.65	*2.08*	0.58	*1.70*	-2.33	*1.80*	-4.64	*1.92*	-4.87	*2.17*	TxA	2.45	0.06
	ZA	39	67.25	*2.26*	0.09	*2.18*	-2.51	*1.90*	-9.50	*2.88*	-3.03	*1.72*	T	0.14	0.71
**Calm**	pl	39	70.06	*2.18*	-0.09	*2.20*	-0.04	*2.37*	0.33	*2.06*	-1.63	*2.47*	TxA	1.23	0.30
	ZA	39	67.62	*2.34*	0.26	*1.51*	-0.74	*1.89*	-3.60	*2.21*	-4.33	*1.73*	T	0.48	0.49
**Content**	pl	39	74.43	*1.93*	-0.63	*1.28*	-2.00	*1.32*	-2.62	*1.34*	-1.97	*1.45*	TxA	1.54	0.21
	ZA	39	74.06	*2.42*	-0.40	*1.89*	-0.85	*1.95*	-5.17	*2.41*	-0.90	*1.63*	T	0.00	0.99

Table S5. Acute Day 1 data for the Cognitive Demand Battery outcomes. Showing raw data for the three pre-dose Day 1 baseline repetitions of the battery and change from baseline data for the 3 repetitions of the Cognitive Demand Battery at each post-dose assessment (all mean plus SEM). The ANOVA columns show the F scores and probabilities from the two-way ANOVA for that outcome: TxA = the treatment x assessment interaction term; T = the main effect of treatment term.

			**Baseline**			**Assessment** (change from baseline data)											
			**Pre-dose**			**1-hour post-dose**			**3-hour post-dose**			**6-hour post-dose**			**ANOVA**		
**outcome**		N	Rep 1	Rep 2	Rep 3	Rep 1	Rep 2	Rep 3	Rep 1	Rep 2	Rep 3	Rep 1	Rep 2	Rep 3		F	P
**Mental**	pl	42	47.79 *2.85*	49.79 *3.29*	58.40 *3.42*	4.86 *2.40*	11.7 *2.47*	6.55 *2.32*	17.36 *2.77*	20.71 *2.92*	14.88 *2.72*	17.79 *3.25*	23.98 *2.86*	19.07 *2.36*	TxA	0.28	0.75
**Fatigue** (mm)	ZA	39	55.95 *3.17*	64.15 *3.02*	70.82 *2.89*	4.39 *2.49*	5.03 *2.56*	0.28 *2.41*	13.18 *2.87*	12.56 *3.03*	10.51 *2.82*	16.51 *3.38*	12.64 *2.97*	12.13 *2.45*	T	3.45	0.07
**RVIP %**	pl	42	58.57 *3.17*	55.06 *3.32*	54.11 *3.49*	-1.4 *2.07*	-1.43 *1.68*	0.66 *1.88*	-2.98 *2.11*	-3.27 *2.02*	-1.79 *2.17*	-5.00 *2.46*	-6.96 *2.08*	-3.04 *2.15*	TxA	0.02	0.97
**correct**	ZA	35	53.36 *3.52*	55.00 *3.14*	56.50 *2.71*	2.71 *2.26*	-2.50 *1.84*	-4.6 *2.06*	1.29 *2.31*	-5.79 *2.22*	-7.14 *2.37*	-2.00 *2.70*	-6.57 *2.28*	-9.29 *2.35*	T	0.27	0.61
**RVIP RT**	pl	42	490.3 *8.16*	497.9 *8.15*	501.9 *8.55*	4.34 *5.54*	9.30 *4.92*	1.41 *5.68*	9.16 *5.37*	10.71 *5.77*	0.54 *6.47*	3.84 *7.00*	-2.49 *6.15*	-4.47 *7.17*	TxA	1.08	0.34
msec	ZA	35	509.4 *7.18*	514.2 *8.11*	528.2 *8.87*	-0.5 *6.07*	3.52 *5.38*	-11.3 *6.23*	-2.92 *5.88*	-6.28 *6.32*	-14.2 *7.08*	-13.4 *7.67*	-7.48 *6.73*	-7.81 *7.86*	T	3.80	0.05
**RVIP FA**	pl	42	2.45 *0.34*	1.57 *0.28*	1.38 *0.19*	-1.45 *0.48*	0.00 *0.29*	0.05 *0.27*	-1.17 *0.50*	0.19 *0.31*	-0.19 *0.27*	-0.98 *0.45*	-0.02 *0.34*	0.24 *0.37*	TxA	0.20	0.82
No.	ZA	35	3.51 *0.72*	3.00 *0.45*	2.51 *0.37*	-1.97 *0.53*	-0.74 *0.32*	-0.63 *0.30*	-1.91 *0.55*	-0.71 *0.34*	-0.34 *0.29*	-1.89 *0.50*	-0.86 *0.38*	-0.34 *0.41*	T	5.25	0.02
**Serial 3**	pl	41	33.15 *2.27*	35.98 *2.41*	36.37 *2.30*	4.44 *0.86*	2.29 *0.96*	1.00 *1.05*	5.63 *1.07*	2.54 *1.02*	1.85 *1.14*	5.12 *1.04*	3.20 *1.08*	2.39 *1.25*	TxA	1.06	0.35
**Correct** No.	ZA	39	32.54 *2.32*	34.15 *2.33*	34.08 *2.41*	4.03 *0.88*	2.80 *0.99*	1.62 *1.08*	4.36 *1.10*	3.08 *1.05*	3.15 *1.17*	4.10 *1.07*	1.05 *1.10*	2.21 *1.28*	T	0.07	0.79
**Serial 3**	pl	41	1.46 *0.27*	2.15 *0.35*	1.80 *0.31*	0.78 *0.38*	0.10 *0.47*	0.37 *0.47*	0.61 *0.43*	-0.56 *0.35*	-0.42 *0.42*	0.63 *0.46*	-0.61 *0.44*	-0.10 *0.42*	TxA	1.09	0.34
**Error** No.	ZA	39	2.15 *0.38*	2.33 *0.37*	2.79 *0.42*	0.59 *0.39*	0.26 *0.48*	0.08 *0.48*	0.90 *0.45*	-0.36 *0.36*	-0.49 *0.43*	0.74 *0.48*	0.59 *0.45*	-0.26 *0.43*	T	0.16	0.69
**Serial 3**	pl	41	34.61 *2.24*	38.12 *2.34*	38.17 *2.29*	5.22 *0.77*	2.39 *0.79*	1.37 *0.91*	6.24 *0.93*	1.98 *0.96*	1.44 *0.99*	5.76 *0.88*	2.59 *0.94*	2.29 *1.19*	TxA	0.79	0.46
**Total** No.	ZA	39	34.69 *2.24*	36.49 *2.33*	36.87 *2.40*	4.62 *0.79*	3.05 *0.81*	1.69 *0.93*	5.26 *0.95*	2.72 *0.98*	2.67 *1.01*	4.85 *0.90*	1.64 *0.97*	1.95 *1.22*	T	0.01	0.91
**Serial 7**	pl	41	20.90 *1.91*	22.83 *2.00*	21.90 *1.84*	2.32 *0.77*	1.49 *0.67*	2.27 *0.80*	4.05 *0.82*	1.46 *0.83*	3.15 *0.91*	4.66 *0.79*	1.51 *0.93*	4.17 *0.85*	TxA	2.33	0.10
**Correct** No.	ZA	39	19.67 *1.67*	21.21 *1.80*	19.69 *1.77*	2.33 *0.79*	1.08 *0.69*	1.44 *0.82*	4.15 *0.84*	1.85 *0.85*	3.87 *0.94*	2.67 *0.81*	1.00 *0.95*	3.28 *0.87*	T	0.24	0.62
**Serial 7**	pl	41	2.39 *0.30*	2.22 *0.34*	2.49 *0.33*	0.12 *0.45*	0.15 *0.35*	0.27 *0.46*	0.00 *0.42*	0.27 *0.57*	-0.71 *0.53*	-0.44 *0.37*	0.49 *0.48*	-0.44 *0.48*	TxA	0.07	0.93
**Error** No.	ZA	39	2.67 *0.44*	2.56 *0.33*	3.90 *0.54*	0.51 *0.46*	0.44 *0.36*	-0.03 *0.47*	-0.08 *0.43*	0.44 *0.58*	-0.51 *0.55*	0.36 *0.38*	0.82 *0.49*	-0.85 *0.50*	T	0.18	0.68
**Serial 7**	pl	41	23.29 *1.85*	25.05 *1.96*	24.39 *1.84*	2.44 *0.68*	1.63 *0.60*	2.54 *0.66*	4.05 *0.69*	1.73 *0.74*	2.44 *0.76*	4.22 *0.72*	2.00 *0.74*	3.73 *0.68*	TxA	2.79	0.06
**Total** No.	ZA	39	22.33 *1.56*	23.77 *1.71*	23.59 *1.63*	2.85 *0.69*	1.51 *0.62*	1.41 *0.67*	4.08 *0.70*	2.28 *0.75*	3.36 *0.78*	3.03 *0.74*	1.82 *0.76*	2.44 *0.70*	T	0.12	0.73

Table S6 (part 1). Chronic Day 56 data for the Cognitive Demand Battery outcomes. Showing raw data for the three pre-dose Day 1 baseline repetitions of the battery (part 1) and change from baseline data for the the 3 repetitions of the Cognitive Demand Battery during the Day 56 pre-dose assessment (part 1) and the 1 hour, 3 hour and 6 hour post-dose assessments (part 2) (all mean plus SEM). The ANOVA columns show the F scores and probabilities from the two-way ANOVA for that outcome: TxA = the treatment x assessment interaction term; T = the main effect of treatment term.

			**Day 1 Baseline**						**Day 56 Assessment** (change from baseline)					
			**Baseline assessment** (pre-dose)						**Pre-dose**					
**outcome**		N	Rep 1		Rep 2		Rep 3		Rep 1		Rep 2		Rep 3	
**Mental Fatigue** (mm)	pl	39	48.1	2.98	49.5	3.36	58.4	*3.52*	-5.54	*3.20*	-1.31	*3.10*	-3.77	*3.39*
	ZA	39	55.9	3.17	64.2	3.02	70.8	*2.89*	-7.13	*3.20*	-10.6	*3.10*	-12.9	*3.39*
**RVIP % correct**	pl	38	58.56	3.33	56.06	3.41	54.75	*3.63*	-2.30	*2.09*	1.91	*2.20*	3.16	*2.55*
	ZA	35	53.36	3.52	55.00	3.14	56.50	*2.71*	5.57	*2.18*	2.29	*2.29*	-2.14	*2.66*
**RVIP RT** msec	pl	38	489.6	8.53	497.7	8.56	499.3	*8.78*	-1.23	*6.55*	0.52	*6.18*	7.24	*7.69*
	ZA	35	509.4	7.18	514.2	8.11	528.2	*8.87*	-12.4	*6.83*	-10.1	*6.44*	-20.6	*8.01*
**RVIP FA** No.	pl	38	2.45	0.34	1.63	0.29	1.40	*0.20*	-0.21	*0.52*	-0.16	*0.32*	-0.32	*0.30*
	ZA	35	3.51	0.72	3.00	0.45	2.51	*0.37*	-1.26	*0.54*	-0.86	*0.33*	-0.31	*0.31*
**Serial 3 Correct** No.	pl	38	33.56	2.37	36.54	2.50	36.90	*2.37*	4.47	*1.06*	3.32	*1.03*	2.95	*1.11*
	ZA	39	32.54	2.32	34.15	2.33	34.08	*2.41*	3.80	*1.04*	3.67	*1.01*	3.59	*1.09*
**Serial 3 Error** No.	pl	38	1.36	0.28	2.13	0.36	1.74	*0.32*	0.00	*0.37*	-0.63	*0.44*	0.17	*0.45*
	ZA	39	2.15	0.38	2.33	0.37	2.79	*0.42*	0.00	*0.37*	0.21	*0.43*	-0.21	*0.45*
**Serial 3 Total** No.	pl	38	34.92	2.35	38.67	2.43	38.64	*2.37*	4.47	*0.97*	2.68	*0.94*	3.12	*1.06*
	ZA	39	34.69	2.24	36.49	2.33	36.87	*2.40*	3.80	*0.96*	3.87	*0.92*	3.39	*1.05*
**Serial 7 Correct** No.	pl	38	21.31	1.97	23.10	2.07	22.33	*1.89*	1.92	*0.81*	1.68	*0.73*	2.58	*0.92*
	ZA	39	19.67	1.67	21.21	1.80	19.69	*1.77*	1.21	*0.80*	1.05	*0.72*	3.05	*0.90*
**Serial 7 Error** No.	pl	38	2.28	0.30	2.18	0.34	2.41	*0.34*	-0.51	*0.44*	-0.16	*0.39*	-0.21	*0.55*
	ZA	39	2.67	0.44	2.56	0.33	3.90	*0.54*	0.44	*0.43*	0.51	*0.39*	-0.82	*0.54*
**Serial 7 Total** No.	pl	38	23.59	1.91	25.28	2.04	24.74	*1.89*	1.41	*0.72*	1.53	*0.68*	2.37	*0.83*
	ZA	39	22.33	1.56	23.77	1.71	23.59	*1.63*	1.64	*0.71*	1.56	*0.67*	2.23	*0.82*

Continued – Table S6. (part 2)….

			**Day 56 Assessment** (change from baseline)																				
			**1-hour post-dose**						**3-hour post-dose**						**6-hour post-dose**						**ANOVA**		
			Rep 1		Rep 2		Rep 3		Rep 1		Rep 2		Rep 3		Rep 1		Rep 2		Rep 3			F	P
**Mental**	pl	39	5.97	*3.12*	13.59	*3.04*	5.77	*3.21*	11.31	*3.20*	16.59	*3.36*	11.87	*3.30*	18.26	*3.12*	22.23	*3.35*	17.82	*3.20*	TxA	1.88	0.13
**Fatigue** (mm)	ZA	39	0.44	*3.12*	-3.05	*3.04*	-6.39	*3.21*	7.95	*3.20*	4.41	*3.36*	1.46	*3.30*	8.10	*3.12*	7.08	*3.35*	2.13	*3.20*	T	8.01	0.01
**RVIP %**	pl	38	-0.07	*2.64*	-0.72	*2.45*	-0.99	*2.52*	-2.96	*2.86*	-3.09	*2.13*	-2.83	*2.37*	-3.75	*2.73*	-4.08	*2.56*	-5.72	*2.69*	TxA	0.87	0.46
**correct**	ZA	35	7.57	*2.75*	1.00	*2.56*	0.71	*2.62*	4.57	*2.98*	-1.36	*2.22*	-3.86	*2.47*	3.00	*2.84*	-4.07	*2.67*	-7.71	*2.81*	T	0.70	0.41
**RVIP RT**	pl	38	2.96	*7.46*	4.55	*6.11*	1.72	*6.31*	1.73	*8.43*	3.74	*6.78*	1.19	*7.20*	5.01	*8.74*	-1.57	*7.41*	-2.44	*6.39*	TxA	3.74	0.06
msec	ZA	35	-12.0	*7.77*	-4.92	*6.36*	-22.1	*6.58*	-11.9	*8.78*	-5.20	*7.06*	-10.6	*7.50*	-2.33	*9.11*	-8.94	*7.72*	-14.5	*6.66*	T	0.82	0.48
**RVIP FA**	pl	38	-1.45	*0.50*	-0.32	*0.35*	-0.21	*0.35*	-1.24	*0.48*	-0.63	*0.36*	-0.21	*0.31*	-1.24	*0.52*	-0.68	*0.40*	-0.11	*0.35*	TxA	3.24	0.08
No.	ZA	35	-1.89	*0.52*	-1.03	*0.36*	-0.80	*0.36*	-2.14	*0.50*	-1.37	*0.37*	-0.77	*0.32*	-2.14	*0.55*	-1.14	*0.42*	-0.51	*0.36*	T	0.11	0.95
**Serial 3**	pl	38	7.74	*1.06*	4.82	*1.17*	4.45	*1.39*	7.03	*1.08*	2.95	*1.09*	2.55	*1.45*	7.76	*0.95*	4.00	*1.28*	3.45	*1.64*	TxA	6.42	0.00
**Correct** No.	ZA	39	7.82	*1.05*	4.90	*1.15*	3.72	*1.37*	8.08	*1.07*	7.49	*1.08*	6.85	*1.44*	6.85	*0.94*	3.92	*1.27*	2.28	*1.62*	T	0.32	0.57
**Serial 3**	pl	38	0.97	*0.55*	-0.21	*0.47*	-0.24	*0.49*	0.74	*0.45*	-0.16	*0.42*	0.61	*0.51*	0.21	*0.47*	-0.42	*0.56*	0.13	*0.67*	TxA	2.11	0.10
**Error** No.	ZA	39	0.92	*0.54*	0.74	*0.47*	0.90	*0.48*	0.69	*0.44*	-0.13	*0.42*	0.03	*0.50*	1.21	*0.46*	0.49	*0.55*	0.41	*0.66*	T	0.73	0.40
**Serial 3**	pl	38	8.71	*1.01*	4.61	*1.13*	4.21	*1.21*	7.76	*1.05*	2.79	*1.09*	3.16	*1.36*	7.97	*0.96*	3.58	*1.17*	3.58	*1.58*	TxA	5.03	0.00
**Total** No.	ZA	39	8.74	*0.99*	5.64	*1.12*	4.62	*1.19*	8.77	*1.03*	7.36	*1.08*	6.87	*1.34*	8.05	*0.95*	4.41	*1.15*	2.69	*1.56*	T	0.73	0.40
**Serial 7**	pl	38	4.18	*0.88*	2.87	*0.70*	3.55	*1.08*	5.45	*0.94*	3.82	*0.91*	3.45	*1.00*	6.13	*0.98*	3.89	*0.89*	3.55	*1.23*	TxA	1.12	0.34
**Correct** No.	ZA	39	3.10	*0.87*	2.74	*0.69*	4.64	*1.06*	5.05	*0.93*	4.36	*0.90*	5.10	*0.99*	5.41	*0.97*	1.85	*0.87*	3.51	*1.22*	T	0.04	0.84
**Serial 7**	pl	38	-0.16	*0.57*	-0.21	*0.52*	0.40	*0.73*	-0.55	*0.41*	-0.42	*0.46*	-0.11	*0.60*	-0.34	*0.49*	-0.18	*0.58*	0.18	*0.89*	TxA	1.24	0.30
**Error** No.	ZA	39	1.67	*0.56*	1.03	*0.51*	-0.26	*0.72*	0.46	*0.41*	0.56	*0.45*	0.13	*0.59*	0.64	*0.49*	1.92	*0.57*	0.82	*0.88*	T	2.00	0.16
**Serial 7**	pl	38	4.03	*0.89*	2.66	*0.67*	3.95	*0.94*	4.90	*0.88*	3.40	*0.86*	3.34	*0.94*	5.79	*0.88*	3.71	*0.82*	3.74	*1.27*	TxA	1.14	0.33
**Total** No.	ZA	39	4.77	*0.88*	3.77	*0.66*	4.39	*0.92*	5.51	*0.86*	4.92	*0.85*	5.23	*0.93*	6.05	*0.87*	3.77	*0.81*	4.33	*1.25*	T	0.48	0.49

Table S7. State-Trait Anxiety Inventory. Showing data (average plus SEM) for the pre-dose assessments on Day 1 and Day 56. The ANOVA columns show the F scores and probabilities from the two-way ANOVA for that outcome: DxT = the day x treatment interaction term; T = the main effect of treatment term.

			Visit				ANOVA		
		N	Day 1		Day 56			F	T
State-Trait Anxiety Inventory (state)	pl	39	29.49	*1.10*	30.38	*1.14*	DxT	0.25	0.62
	ZA	40	31.78	*1.24*	31.93	*1.06*	T	1.804	0.183

Table S8. Acute effects of ZA on cerebral blood-flow parameters (total haemoglobin, deoxygenated haemoglobin, oxygenated haemoglobin, % oxygen saturation) on Day 1. Data are means (plus SEM). Pre-dose baseline data and data from each individual post-dose epoch are presented. The probability from the planned comparisons (P) between treatments at each epoch are also shown.

		Total Haemoglobin (µM)					Deoxygenated Haemoglobin (µM)					Oxygenated haemoglobin (µM)					% oxygen saturation				
		placebo		ZA		P	placebo		ZA		P	placebo		ZA		P	placebo		ZA		P
	**Pre-dose Baseline**	35.7	*1.50*	40.52	*2.10*		12.6	*0.59*	13.60	*0.83*		23.07	*1.38*	26.92	*1.48*		64.70	*1.28*	66.30	*1.24*	
**Post-dose task epoch** (change from baseline)	**Rest 1**	-0.27	*0.85*	-0.49	*0.83*	ns	0.31	*0.30*	0.07	*0.29*	ns	-0.58	*0.65*	-0.55	*0.64*	ns	-0.95	*0.47*	-0.63	*0.46*	ns
	**Rest 2**	-0.39	*0.87*	-0.57	*0.85*	ns	0.28	*0.28*	-0.08	*0.27*	ns	-0.67	*0.67*	-0.49	*0.65*	ns	-1.01	*0.43*	-0.30	*0.42*	0.002
	**3s**	-0.04	*0.92*	-0.62	*0.89*	< 0.001	0.25	*0.31*	-0.09	*0.30*	ns	-0.29	*0.70*	-0.54	*0.68*	ns	-0.63	*0.49*	-0.39	*0.48*	ns
	**7s**	0.06	*0.94*	-0.53	*0.92*	< 0.001	0.06	*0.32*	-0.23	*0.32*	ns	0.00	*0.72*	-0.29	*0.70*	ns	-0.03	*0.51*	0.05	*0.50*	ns
	**RVIP 1**	0.00	*0.93*	-0.94	*0.91*	< 0.001	-0.06	*0.32*	-0.29	*0.31*	ns	0.07	*0.69*	-0.66	*0.67*	< 0.001	0.24	*0.46*	-0.17	*0.45*	ns
	**RVIP 2**	0.00	*0.93*	-0.96	*0.91*	< 0.001	-0.12	*0.33*	-0.30	*0.32*	ns	0.13	*0.68*	-0.66	*0.67*	< 0.001	0.38	*0.47*	-0.15	*0.46*	ns
	**3s**	0.02	*0.95*	-0.61	*0.93*	< 0.001	-0.02	*0.32*	-0.35	*0.31*	ns	0.04	*0.72*	-0.26	*0.70*	ns	0.18	*0.50*	0.21	*0.49*	ns
	**7s**	0.02	*0.96*	-0.65	*0.93*	< 0.001	-0.09	*0.33*	-0.41	*0.32*	ns	0.11	*0.73*	-0.24	*0.71*	ns	0.31	*0.51*	0.34	*0.50*	ns
	**RVIP 1**	-0.04	*0.96*	-0.80	*0.93*	< 0.001	-0.22	*0.35*	-0.57	*0.34*	ns	0.18	*0.71*	-0.23	*0.69*	ns	0.60	*0.54*	0.58	*0.53*	ns
	**RVIP 2**	0.04	*1.00*	-0.91	*0.97*	< 0.001	-0.19	*0.36*	-0.49	*0.35*	ns	0.24	*0.73*	-0.42	*0.71*	< 0.001	0.59	*0.54*	0.32	*0.52*	ns
	**3s**	0.12	*0.95*	-0.42	*0.92*	< 0.001	0.00	*0.31*	-0.39	*0.31*	ns	0.12	*0.74*	-0.03	*0.72*	ns	0.21	*0.56*	0.48	*0.55*	ns
	**7s**	0.14	*0.99*	-0.33	*0.97*	0.01	-0.04	*0.33*	-0.39	*0.32*	ns	0.17	*0.78*	0.06	*0.76*	ns	0.33	*0.58*	0.57	*0.56*	ns
	**RVIP 1**	0.13	*0.99*	-0.67	*0.96*	< 0.001	-0.23	*0.34*	-0.47	*0.33*	ns	0.36	*0.75*	-0.19	*0.73*	0.002	0.82	*0.55*	0.53	*0.54*	ns
	**RVIP 2**	0.10	*1.01*	-0.82	*0.98*	< 0.001	-0.24	*0.35*	-0.54	*0.34*	ns	0.34	*0.77*	-0.28	*0.75*	< 0.001	0.79	*0.55*	0.57	*0.54*	ns

Table S9. Global effects of ZA on cerebral blood-flow parameters (total haemoglobin, deoxygenated haemoglobin, oxygenated haemoglobin, % oxygen saturation). Pre-dose and post-dose resting raw data (mean plus SEM) from Day 1 and Day 56 are presented. The ANOVA columns show the F scores and probabilities from the two-way ANOVA for each outcome: DxT = the day x treatment interaction term; T = the main effect of treatment term.

		Day 1				Day 56				ANOVA		
		Pre-dose		Post-dose		Pre-dose		Post-dose			F	P
**Deoxygenated Haemoglobin**	placebo	12.16	*0.73*	12.42	*0.65*	12.11	*0.71*	12.42	*0.66*	DxT	0.28	0.60
	ZA	13.50	*0.71*	13.55	*0.64*	13.20	*0.70*	13.35	*0.64*	T	1.54	0.22
**Oxygenated haemoglobin**	placebo	23.47	*1.55*	22.79	*1.32*	24.47	*1.63*	23.47	*1.35*	DxT	2.18	0.15
	ZA	27.00	*1.51*	26.30	*1.29*	26.35	*1.59*	25.40	*1.32*	T	1.99	0.17
**Total Haemoglobin**	placebo	35.47	*2.01*	35.21	*1.72*	36.58	*2.12*	35.79	*1.82*	DxT	2.47	0.12
	ZA	40.50	*1.96*	39.95	*1.67*	39.55	*2.06*	38.90	*1.77*	T	2.44	0.13
**% oxygen saturation**	placebo	64.79	*1.28*	63.89	*1.21*	66.11	*1.29*	64.89	*1.13*	DxT	2.14	0.15
	ZA	66.30	*1.25*	65.75	*1.18*	65.90	*1.25*	65.15	*1.11*	T	0.29	0.59

Table S10. Acute and chronic effects of ZA on haemodynamic responses to task performance. *Mean (plus SEM) data for each cerebral blood-flow parameter (total haemoglobin, deoxygenated haemoglobin, oxygenated haemoglobin, % oxygen saturation) for the immediately pre-task resting baseline and for each task performance epoch are presented. The probability from the planned comparisons (P) between treatments at each epoch are also shown.*

		**Oxygenated haemoglobin**					**Deoxygenated Haemoglobin**					**Total Haemoglobin**					**% oxygen saturation**				
		Placebo		ZA		P	Placebo		ZA		P	Placebo		ZA		P	Placebo		ZA		P
**DAY 1**																					
**Resting baseline**		22.79	*1.32*	26.30	*1.29*		12.42	*0.65*	13.55	*0.64*		35.21	*1.72*	39.95	*1.67*		63.89	*1.21*	65.75	*1.18*	
**Task epoch** (change from resting baseline data)	**3s**	0.28	*0.15*	-0.02	*0.16*	ns	0.01	*0.07*	-0.08	*0.07*	ns	0.28	*0.15*	-0.10	*0.16*	0.006	0.23	*0.19*	-0.03	*0.17*	ns
	**7s**	0.53	*0.21*	0.23	*0.22*	ns	-0.19	*0.09*	-0.23	*0.09*	ns	0.34	*0.19*	0.00	*0.20*	0.031	0.78	*0.26*	0.29	*0.24*	ns
	**RVIP 1**	0.59	*0.20*	-0.13	*0.20*	<0.001	-0.30	*0.10*	-0.28	*0.10*	ns	0.30	*0.18*	-0.42	*0.18*	<0.001	1.04	*0.26*	0.57	*0.21*	<0.001
	**RVIP 2**	0.68	*0.23*	-0.14	*0.23*	<0.001	-0.35	*0.10*	-0.30	*0.11*	ns	0.33	*0.22*	-0.43	*0.22*	<0.001	1.20	*0.29*	0.68	*0.24*	<0.001
	**3s**	0.52	*0.26*	0.26	*0.26*	ns	-0.29	*0.11*	-0.35	*0.11*	ns	0.23	*0.24*	-0.09	*0.25*	0.050	0.94	*0.31*	0.59	*0.30*	ns
	**7s**	0.62	*0.25*	0.28	*0.26*	0.039	-0.36	*0.11*	-0.40	*0.12*	ns	0.26	*0.25*	-0.12	*0.26*	0.005	1.13	*0.31*	0.67	*0.31*	ns
	**RVIP 1**	0.74	*0.24*	0.29	*0.24*	0.001	-0.46	*0.14*	-0.57	*0.15*	ns	0.28	*0.24*	-0.28	*0.25*	<0.001	1.45	*0.33*	0.85	*0.32*	ns
	**RVIP 2**	0.79	*0.24*	0.10	*0.24*	<0.001	-0.43	*0.14*	-0.49	*0.14*	ns	0.36	*0.25*	-0.38	*0.26*	<0.001	1.44	*0.32*	0.94	*0.30*	<0.001
	**3s**	0.62	*0.27*	0.49	*0.28*	ns	-0.29	*0.10*	-0.39	*0.11*	ns	0.33	*0.26*	0.10	*0.26*	ns	1.05	*0.33*	0.61	*0.32*	ns
	**7s**	0.68	*0.32*	0.59	*0.33*	ns	-0.33	*0.12*	-0.39	*0.12*	ns	0.35	*0.32*	0.20	*0.33*	ns	1.16	*0.36*	0.66	*0.38*	ns
	**RVIP 1**	0.90	*0.29*	0.33	*0.30*	<0.001	-0.48	*0.11*	-0.47	*0.12*	ns	0.42	*0.31*	-0.14	*0.31*	<0.001	1.66	*0.31*	0.90	*0.32*	<0.001
	**RVIP 2**	0.88	*0.29*	0.24	*0.30*	<0.001	-0.50	*0.12*	-0.54	*0.12*	ns	0.38	*0.29*	-0.30	*0.30*	<0.001	1.67	*0.34*	1.00	*0.30*	<0.001
**DAY 56**																					
**Resting baseline**		23.47	*1.35*	25.40	*1.32*		12.42	*0.66*	13.35	*0.64*		35.79	*1.82*	38.90	*1.77*		64.89	*1.13*	65.15	*1.11*	
**Task epoch** (change from resting baseline data)	**3s**	0.12	*0.14*	-0.04	*0.14*	ns	0.04	*0.07*	-0.09	*0.07*	ns	0.16	*0.15*	-0.13	*0.15*	ns	0.07	*0.19*	0.07	*0.17*	ns
	**7s**	0.27	*0.19*	0.12	*0.19*	ns	-0.09	*0.09*	-0.30	*0.09*	0.001	0.19	*0.17*	-0.19	*0.17*	0.007	0.51	*0.27*	0.55	*0.25*	ns
	**RVIP 1**	0.38	*0.15*	-0.20	*0.15*	<0.001	-0.19	*0.09*	-0.20	*0.09*	ns	0.19	*0.14*	-0.40	*0.14*	<0.001	0.29	*0.27*	0.10	*0.22*	0.023
	**RVIP 2**	0.43	*0.19*	-0.24	*0.19*	<0.001	-0.25	*0.09*	-0.18	*0.09*	ns	0.17	*0.19*	-0.41	*0.19*	<0.001	0.31	*0.29*	0.01	*0.24*	<0.001
	**3s**	0.40	*0.24*	-0.10	*0.25*	<0.001	-0.22	*0.11*	-0.24	*0.11*	ns	0.19	*0.22*	-0.33	*0.23*	<0.001	0.67	*0.32*	0.18	*0.31*	ns
	**7s**	0.42	*0.25*	-0.08	*0.25*	<0.001	-0.27	*0.12*	-0.35	*0.12*	ns	0.15	*0.23*	-0.43	*0.23*	<0.001	0.80	*0.31*	0.45	*0.32*	ns
	**RVIP 1**	0.53	*0.25*	-0.14	*0.25*	<0.001	-0.30	*0.12*	-0.29	*0.12*	ns	0.23	*0.20*	-0.43	*0.21*	<0.001	1.04	*0.34*	0.31	*0.33*	0.003
	**RVIP 2**	0.58	*0.25*	-0.15	*0.25*	<0.001	-0.32	*0.11*	-0.28	*0.11*	ns	0.25	*0.23*	-0.43	*0.23*	<0.001	0.79	*0.33*	0.26	*0.31*	<0.001
	**3s**	0.46	*0.26*	-0.03	*0.27*	<0.001	-0.21	*0.11*	-0.22	*0.12*	ns	0.24	*0.23*	-0.25	*0.24*	<0.001	0.94	*0.34*	0.28	*0.33*	ns
	**7s**	0.51	*0.31*	0.03	*0.32*	<0.001	-0.23	*0.14*	-0.27	*0.14*	ns	0.28	*0.26*	-0.23	*0.27*	<0.001	1.03	*0.37*	0.40	*0.39*	ns
	**RVIP 1**	0.61	*0.25*	-0.09	*0.26*	<0.001	-0.30	*0.12*	-0.21	*0.12*	ns	0.31	*0.23*	-0.30	*0.24*	<0.001	0.99	*0.32*	0.25	*0.33*	<0.001
	**RVIP 2**	0.70	*0.28*	-0.10	*0.29*	<0.001	-0.30	*0.11*	-0.24	*0.12*	ns	0.40	*0.30*	-0.35	*0.30*	<0.001	1.03	*0.35*	0.29	*0.31*	<0.001

Table S11. Data for the Cognitive Demand Battery outcomes during the qNIRS assessment on Day 1 (all mean plus SEM). Showing raw data for the three pre-dose Day 1 baseline repetitions of the battery (undertaken as part of the cognitive assessment) and the three repetitions of the Cognitive Demand Battery during the post-dose qNIRS assessment.

			**Assessment**											
			**Day 1 Baseline** (unadjusted)						**2 hours post-dose on Day 1** (change from baseline)					
			Repetition 1		*Repetition 2*		*Repetition 3*		*Repetition 1*		*Repetition 2*		*Repetition 3*	
**Mental Fatigue** (mm)	pl	22	47.23	*4.25*	49.00	*4.69*	57.14	*5.31*	9.36	*4.70*	10.68	*4.17*	3.14	*4.53*
	ZA	20	55.85	*4.09*	62.00	*4.41*	67.60	*4.20*	3.10	*4.93*	0.95	*4.38*	-5.15	*4.75*
**RVIP % correct**	pl	22	57.95	*4.40*	55.11	*4.26*	53.18	*4.75*	-1.82	*2.77*	-0.23	*3.26*	1.02	*3.17*
	ZA	19	60.00	*3.94*	59.74	*3.74*	60.26	*3.18*	4.47	*2.98*	0.66	*3.51*	-1.18	*3.41*
**RVIP RT** msec	pl	22	493.3	*11.5*	487.7	*12.3*	492.2	*11.7*	-25.6	*18.5*	-22.2	*18.9*	-25.9	*17.3*
	ZA	19	507.8	*9.0*	513.9	*9.6*	523.1	*10.5*	-5.6	*19.9*	-5.9	*20.3*	-16.2	*18.6*
**RVIP FA** No.	pl	22	2.41	*0.51*	1.45	*0.41*	1.45	*0.28*	-1.68	*0.47*	-0.59	*0.40*	-0.05	*0.46*
	ZA	19	2.58	*0.67*	3.05	*0.51*	2.11	*0.40*	-0.58	*0.51*	-1.84	*0.43*	-0.95	*0.50*
**Serial 3 Correct** No.	pl	21	33.05	*2.52*	35.00	*2.62*	35.52	*2.05*	4.14	*1.52*	4.05	*0.91*	2.71	*1.46*
	ZA	20	36.95	*3.47*	39.10	*3.38*	38.70	*3.38*	2.90	*1.56*	0.80	*0.94*	1.55	*1.50*
**Serial 3 Error** No.	pl	21	1.38	*0.36*	2.19	*0.48*	1.76	*0.45*	0.43	*0.63*	-0.62	*0.61*	0.81	*0.59*
	ZA	20	2.00	*0.48*	2.30	*0.43*	3.20	*0.59*	0.50	*0.65*	-0.05	*0.62*	-1.15	*0.60*
**Serial 3 Total** No.	pl	21	34.43	*2.57*	37.19	*2.56*	37.29	*2.12*	4.57	*1.42*	3.43	*0.82*	3.52	*1.30*
	ZA	20	38.95	*3.32*	41.40	*3.27*	41.90	*3.35*	3.40	*1.45*	0.75	*0.84*	0.40	*1.33*
**Serial 7 Correct** No.	pl	21	20.62	*2.13*	22.76	*2.36*	21.48	*2.19*	3.38	*1.19*	1.86	*0.90*	2.62	*1.30*
	ZA	20	22.35	*2.34*	23.90	*2.59*	21.75	*2.48*	4.15	*1.22*	3.30	*0.92*	3.60	*1.33*
**Serial 7 Error** No.	pl	21	2.14	*0.32*	2.05	*0.38*	2.38	*0.49*	0.19	*0.56*	0.48	*0.48*	0.10	*0.64*
	ZA	20	3.00	*0.58*	2.85	*0.36*	4.15	*0.82*	-0.45	*0.58*	-0.25	*0.50*	-1.15	*0.66*
**Serial 7 Total** No.	pl	21	22.76	*2.07*	24.81	*2.38*	23.86	*2.15*	3.57	*0.98*	2.33	*0.87*	2.71	*1.11*
	ZA	20	25.35	*2.15*	26.75	*2.49*	25.90	*2.20*	3.70	*1.01*	3.05	*0.89*	2.45	*1.14*

Table S12. Data for the Cognitive Demand Battery outcomes during the qNIRS assessment on Day 56 (all mean plus SEM). Showing raw data for the three pre-dose Day 1 baseline repetitions of the battery (undertaken as part of the cognitive assessment) and the three repetitions of the Cognitive Demand Battery during the post-dose qNIRS assessment on Day 56.

			**Assessment**											
			**Day 1 Baseline** (unadjusted)						**2 hours post-dose on Day 1** (change from baseline)					
			Repetition 1		*Repetition 2*		*Repetition 3*		*Repetition 1*		*Repetition 2*		*Repetition 3*	
**Mental Fatigue** (mm)	pl	21	45.76	*4.18*	46.76	*4.33*	55.14	*5.16*	4.67	*4.17*	5.10	*4.11*	-0.24	*5.36*
	ZA	19	55.26	*4.27*	61.42	*4.61*	67.05	*4.39*	0.58	*4.38*	0.58	*4.32*	-2.05	*5.63*
**RVIP % correct**	pl	21	59.17	*4.44*	55.71	*4.43*	53.33	*4.97*	1.55	*3.89*	1.19	*3.33*	-0.48	*3.53*
	ZA	18	59.31	*4.10*	60.14	*3.93*	60.42	*3.35*	6.67	*4.20*	6.11	*3.59*	0.14	*3.82*
**RVIP RT** msec	pl	21	492.4	*12.0*	486.4	*12.8*	492.2	*12.3*	-9.6	*9.9*	0.0	*7.3*	1.1	*8.9*
	ZA	18	507.2	*9.5*	510.2	*9.4*	520.6	*10.8*	-2.2	*10.7*	-8.4	*7.9*	-18.9	*9.6*
**RVIP FA** No.	pl	21	2.00	*0.32*	1.19	*0.33*	1.43	*0.29*	-1.29	*0.51*	-0.43	*0.46*	-0.48	*0.35*
	ZA	18	2.72	*0.69*	3.22	*0.51*	2.22	*0.41*	-1.78	*0.55*	-1.89	*0.50*	-0.61	*0.38*
**Serial 3 Correct** No.	pl	20	33.60	*2.58*	35.40	*2.72*	36.05	*2.08*	5.75	*1.39*	5.15	*1.15*	4.85	*1.62*
	ZA	19	37.00	*3.65*	39.53	*3.53*	38.84	*3.56*	5.79	*1.43*	3.90	*1.18*	4.53	*1.66*
**Serial 3 Error** No.	pl	20	1.45	*0.37*	2.30	*0.49*	1.75	*0.48*	0.85	*0.71*	-0.30	*0.61*	-0.30	*0.53*
	ZA	19	2.11	*0.49*	2.32	*0.45*	3.32	*0.61*	0.68	*0.73*	0.05	*0.63*	-1.05	*0.55*
**Serial 3 Total** No.	pl	20	35.05	*2.62*	37.70	*2.64*	37.80	*2.16*	6.60	*1.29*	4.85	*1.21*	4.55	*1.68*
	ZA	19	39.11	*3.49*	41.84	*3.41*	42.16	*3.52*	6.47	*1.33*	3.95	*1.24*	3.47	*1.72*
**Serial 7 Correct** No.	pl	20	21.25	*2.14*	23.30	*2.42*	21.85	*2.27*	6.75	*1.23*	4.60	*0.99*	5.30	*1.38*
	ZA	19	22.37	*2.47*	24.16	*2.72*	21.74	*2.62*	6.79	*1.26*	4.95	*1.02*	4.95	*1.42*
**Serial 7 Error** No.	pl	20	2.20	*0.33*	2.05	*0.40*	2.50	*0.49*	-0.70	*0.57*	-0.10	*0.55*	-0.85	*0.72*
	ZA	19	3.16	*0.59*	2.84	*0.38*	4.32	*0.85*	-0.26	*0.59*	0.00	*0.56*	-0.63	*0.74*
**Serial 7 Total** No.	pl	20	23.45	*2.06*	25.35	*2.44*	24.35	*2.20*	6.05	*0.96*	4.50	*1.00*	4.45	*1.12*
	ZA	19	25.53	*2.26*	27.00	*2.61*	26.05	*2.31*	6.53	*0.99*	4.95	*1.03*	4.32	*1.15*
